# Encoding Collective Knowledge, Instructing Data Reusers: The Collaborative Fixation of a Digital Scientific Data Set

**DOI:** 10.1007/s10606-021-09407-2

**Published:** 2021-10-25

**Authors:** Götz Hoeppe

**Affiliations:** Departments of Anthropology and Sociology & Legal Studies, University of Waterloo, 200 University Avenue West, Waterloo, Ontario N2L 3G1 Canada

**Keywords:** Algorithms, Astronomy, Collective knowledge, Collaborative research practices, Coordinative artifacts, Data sharing, Data reuse, Ethnomethodology, Testing

## Abstract

This article provides a novel perspective on the use and reuse of scientific data by providing a chronological ethnographic account and analysis of how a team of researchers prepared an astronomical catalogue (a table of measured properties of galaxies) for public release. Whereas much existing work on data reuse has focused on information about data (such as metadata), whose form or lack has been described as a hurdle for reusing data successfully, I describe how data makers tried to instruct users through the processed data themselves. The fixation of this catalogue was a negotiation, resulting in what was acceptable to team members and coherent with the diverse data uses pertinent to their completed work. It was through preparing their catalogue as an ‘instructing data object’ that this team seeked to encode its members’ knowledge of how the data were processed and to make it consequential for users by devising methodical ways to structure anticipated uses. These methods included introducing redundancies that would help users to self-correct mistaken uses, selectively deleting data, and deflecting accountability through making notational choices. They dwell on an understanding of knowledge not as exclusively propositional (such as the belief in propositions), but as embedded in witnessable activities and the products of these activities. I discuss the implications of this account for philosophical notions of collective knowledge and for theorizing coordinative artifacts in CSCW. Eventually, I identify a tension between ‘using algorithms’ and ‘doing science’ in preparing data sets and show how it was resolved in this case.

## Introduction

Large digital scientific data sets are central to the work of diverse scientific disciplines (Borgman 2015; Edwards 2010; Hilgartner 2017; Hoeppe 2014, 2020; Leonelli 2016). Typically generated by large teams, large data sets usually include processed, higher-level data, such as measurements, that are useful for diverse scientific studies.^1^ Often made using public facilities and in the course of projects that are financed with tax money, many large data sets become available through open access (Borgman 2015; Leonelli 2016). This reflects both institutional mandates as well as scientists’ widespread and increasing endorsement, support, and expectation to share research data (Curty et al. 2017; Kratz and Strasser 2015; Tenopir et al. 2011, 2015).

For scientists to collaborate in making and releasing a large digital data set for the successful reuse by other scientists is a complex process that is literally possible only through computer-supported cooperative work. Several threads of research in CSCW and Science and Technology Studies (STS) help to make sense of such an undertaking and its challenges. A team needs to structure and coordinate its activities (Bietz et al. 2010; Paine and Lee 2021; Wulf 1993), and this involves articulation work (Strauss 1988), including the division, allocation, coordination, scheduling, meshing and interrelating of ‘distributed individual activities’ (Schmidt and Bannon 1992, p. 14). This is supported, shaped and challenged by infrastructures (Jirotka et al. 2013). Temporal aspects are critical (Steinhardt and Jackson 2014), and actors often ‘interact “through” a collection of artifacts of various kinds’ (Schmidt and Wagner 2004, p. 350; cf. Lee 2007), including coordinative artifacts – ‘stable data structure[s] expressed in a standardized graphical format’ (Schmidt 2011, p. 16). Large data sets are typically made available, and accessed, through databases (Bietz and Lee 2009; Leonelli 2016).

It has been claimed that for its reuse^2^ to succeed, scientific data need to be findable, accessible, interoperable and reusable (FAIR, Wilkinson et al. 2016). Yet with data being released there come to be makers and (potential) reusers, and along with this come matters of communication, understanding, proper use, and social accountability. Will users understand our data? Do data producers share all the information we need? Will someone be blamed? And if so, who, and by whom?

Finding existing data and making it usable in the absence of personal communications can be challenging, since data reusers often need to know more than metadata (‘data describing data’) provide. Instead of regarding metadata as fixed products, this ‘metadata friction’ can sometimes be resolved through a process of communication, in which data reusers seek to establish common ground (Clark 1996) with data producers (Edwards et al. 2011; cf. also Mayernik 2019). Reusers may experience similar problems with the data themselves. Successful reuses often hinge on an understanding of the context of data generation that may not be knowable from the available data and metadata (Birnholtz and Bietz 2003; Chin and Lansing 2004; Carlson and Anderson 2007). Reusers may seek to gain this information through personal contacts (Faniel and Jacobsen 2010) or may draw on their own disciplinary training to make educated guesses about what data makers could have done (Zimmerman 2007). Assessing the usability and reliability of data often turns out to be a protracted and iterative process (Rolland and Lee 2013), prompting efforts to typify the desired contextual information (Faniel et al. 2019; Yakel et al. 2019).

Attempts to reuse data may fail (Yoon 2016), and scientists worry about being held accountable for wrong uses of their data, fearing a dent to their reputation and credibility (Brewer 2017). Making the diverse contextual information that reusers desire explicit can be an overwhelming task for data makers (cf. Faniel et al. 2019; Yakel et al. 2019). Borrowing insights from studies of design it seems conceivable that data makers would instead seek to ‘configure’ data users (Woolgar 1991), ‘script’ users into the design (Akrich 1992), or consider users as ‘scenic features’ (Sharrock and Anderson 1994) and introduce them into the structure of their data releases as typifications, or as ‘contexts’ of design (Martin et al. 2007). Makers of data may, of course, provide instructions or user manuals along with their data. In fact, scientific data releases are often accompanied by papers that describe the data production, reduction and analysis (Pasquetto et al. 2017). These papers are typically meant to instruct users. But can such instructions be complete, and will data users follow them? After all, studies of technology use have provided ample demonstrations that many users do not consult manuals when setting out to operate new devices, or turn to them only when troubles are obvious (Novick and Ward 2006; Blackler et al. 2016).

Such attitudes have inspired designers of machines to develop artifacts that aspire to be ‘self-explanatory’, that is, ‘their operation should be discoverable without extensive training, from information provided on or through the machine itself’ (Suchman 2007, p. 43). Yet even when users try to follow such instructions they are bound to be challenged. Suchman’s (2007) study of how users of a photocopy machine interact with its support system, which she had based on investigating talk-in-interaction, builds on, and illustrates, Garfinkel’s (1967) insight that all instructions are essentially incomplete and context-dependent(cf. Lindwall et al. 2015). This finding extends to scientific practice (Lynch and Jordan 1995) and it is likely that it will pertain to data reuses as well.

This paper addresses these challenges. It provides a novel perspective on the release of scientific data sets by offering a ethnographic account and analysis of how a team of scientists prepared and released an astronomical catalogue, a higher-level data product. Made by MUWAGS (Multi-Wavelength Galaxy Survey; pseudo-acronym), a team of 30 astronomers from 10 countries, this was a large table (90,000 rows and 200 columns) of measurements of objects in a certain part of the sky, including their celestial coordinates, classification (e.g. ‘star’ or ‘galaxy’) and physical parameters such as brightness, colours, distance, and mass. It was intended both for the team’s own future work as well as for uses by other researchers. The team could have prepared their data in many different ways. So why did they release it just like they did?^3^ I argue that generalizable insights can be gained from considering this case in detail.

I wish to make three interventions to the CSCW and STS literature on the use and reuse of scientific data. First, whereas existing work on data reuse has focused on knowledge and information *about data* (e.g. Edwards et al. 2011; Faniel et al. 2019; Mayernik 2019), which has often prompted reusers to try to contact data makers, I demonstrate how data makers may try to instruct users *through the data* themselves, and seek to create conditions for making communications with users unnecessary. Whereas Faniel et al. (2019) emphasize data reusers’ common need for making contexts of data production explicit in detail, I consider situations in which scientists try to work their practical knowledge of this context into their data.

This calls, secondly, for a refined consideration of how the processing of data is embedded in the professional ‘form of life’ (Wittgenstein 2009) of their makers. Bowker (2005, p. 184) argued that, due to the unavoidably context-dependent nature of data production, there are no ‘raw’ data (cf. also Mosconi et al. 2019). However, a meaningful distinction can be made between ‘primary’ data – unprocessed outputs of data generators or instrumental recordings that have been variously called ‘inscriptions’ (Latour and Woolgar 1986), ‘data’ (Hacking 1992) or ‘traces’ (Rheinberger 2011)– and the processed and calibrated outputs useful for the production of a scientific result – for which Rheinberger (2011), for example, reserves the term ‘data’. One can think of this distinction as the endpoints of a continuum. The latter are not only more ‘theory-laden’ (Hanson 1958) than unprocessed instrumental recordings, but also more ‘practice-laden’ (see Section 2.2).

Thirdly, I argue that it is through fixating a data release into what I call an ‘instructing data object’ that a team may seek to encode its collective knowledge of data processing and make it consequential for further uses. This process is a negotiation that, if successful, results in a product that is acceptable to team members and coherent with the diverse uses pertinent to their completed work. The released data product is bound to have a formatted structure, that, like coordinative artifacts, may encompass a variety of substrates including digital files and paper documents (Anderson and Sharrock 2018; Schmidt and Wagner 2004). Yet, taken on their own, formatted structures do not guarantee alignment. Their uses ought to be situated in shared practices (Goodwin 2013, 2018; Harper 1998), reminiscent of the coordination mechanisms considered in CSCW (Schmidt and Simone 1996). Suchman’s (2007, p. 192) wariness of the limits of inscribing users and uses into design is bound to apply to scientific data releases and other instructing data objects.

Focusing on the practical reasoning and the actions of scientists, my analysis is informed by ethnomethodology (Garfinkel 1967, 2002; Randall et al. 2007). Instructed action, from assembling mail-order furniture to operating machines, has been a major focus of ethnomethodological studies (Garfinkel 2002; Suchman 2007; Lindwall et al. 2015, and references therein). I inquire into the perspective of members and address how they have resolved technical issues to their satisfaction. In doing so I attend to the temporality and sequentiality of their work and to how they made their actions accountable, that is, witnessable and reportable (Garfinkel 1967, p. 1).^4^ As I consider the *collaborative* fixation^5^ of a digital object, team members’ mutual understandings, as revealed by their interactions, are critical to my account. I draw on detailed transcriptions of selected conversational exchanges to reveal team members’ practical reasoning and the methods they used to instruct users through the catalogue. Although I use elements of conversation analysis to describe and analyse several audio-recorded interactions this is not a study of conversation analysis.

I begin with a brief introduction to astronomical catalogues, and practical issues commonly encountered in their fixation (Section 2). Then I give a chronological account of how the MUWAGS team prepared its catalogue, focusing on several moments in which they made critical decisions (Section 3). While this work is ostensibly oriented to outsiders, the process of achieving an agreement on which data to release, and how to release them, required team members to negotiate what the catalogue was meant to be for themselves as well. As such the discussion must return to catalogues as instructing data objects, the materialisation of collective knowledge in the data release, and a tension (identified in Section 2) between ‘using algorithms’ and ‘doing science’ in the work of distributed collaborations of scientists (Section 4).

This paper draws on my ethnography of the MUWAGS team. For about 12 months I was a daily visitor of a working group that included a sub-team of the MUWAGS collaboration, following the researchers’ work day. I witnessed data analysis work, instructional meetings, team meetings and teleconferences, conducted interviews, accessed their emails and assisted in a small part of their research. For doing so I benefitted from my own graduate training in astrophysics. I witnessed most of the team discussions relating to the public data release. This text draws on my field notes, 492 emails pertaining to the data release, as well as transcribed audio recordings of 7 interviews, 10 teleconferences, as well as 56 recordings made at four collaboration meetings.

Even though the work that I witnessed mostly happened in 2007 and 2008, my re-visits to the field in 2010–2019 and ongoing conversations indicate its enduring relevance. I use pseudonyms for all the conversationalists whom I quote or refer to in this text, and pseudo-acronyms in referring to their collaboration and the object studied. Trancriptions of recorded interviews are set in normal text font, those of recorded naturally occurring conversations are set in Courier New.^6^

## Astronomical catalogues and the challenges of their fixation

### Astronomical catalogues

Catalogues – lists or tables of the measured or estimated properties of celestial objects – are a dominant form of data in astronomy (Djorgovski et al. 2013; Jaschek 1984; Ochsenbein et al. 2000). Traditionally, catalogues include objects of a specific kind, such as stars, gas nebulae and galaxies. For example, the list of the positions of 850 stars measured by the Greek astronomer Hipparchus ca. 130 BCE is called the Hipparchus catalogue. It is the oldest extant astronomical catalogue. Many contemporary catalogues also contain data on a single kind of object, but others include the measured properties of diverse objects, such as all those detected with a certain detector attached to a specific telescope in the course of a research project. Sky surveys are projects in which objects in a large part of the sky are systematically observed and measured with a specific set-up of telescope, detector and filter-set (transparent to specific wavelengths of light), and are processed with specific computer code. Many of the largest catalogues result from surveys, comprising the work of dozens to hundreds of scientists and technical experts (Djorgovski et al. 2013). Thus, the early Data Release 3 (eDR3) of the European Space Agency’s *Gaia* satellite mission, published in December 2020, contains measurements of the position and brightness of 1.8 billion celestial sources, most of which are stars in our Galaxy (Gaia Collaboration 2021). Its data release paper lists 422 co-authors. The final *Gaia* data release, expected for the mid-2020s, is bound to be yet larger. At the time of writing (June 11, 2021), the Centre des Données Astronomiques de Strasbourg (France), a major astronomical data center, provides digital access to 20,945 astronomical catalogues.^7^

**Figure 1.**
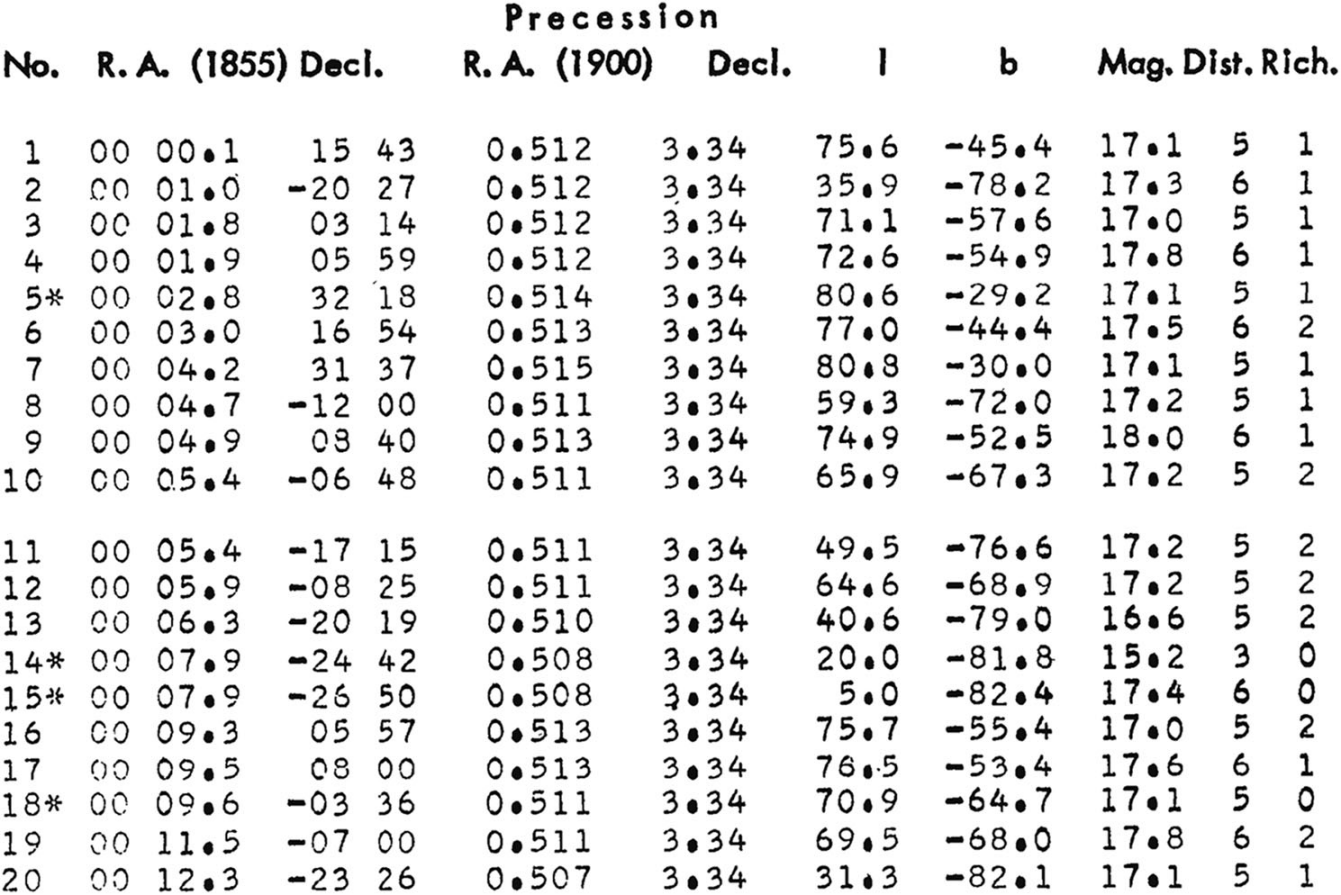
The first entries of George Abell’s (1958) catalogue of 2712 galaxy clusters in the northern and equatorial sky, based on his visual inspection of the photographic plates of the Palomar Observatory Sky Survey. For each object the columns list the catalogue numbers (column 1), celestial coordinates and positional information (columns 2 to 7), the visually estimated magnitude of the 10th brightest cluster galaxy (columns 8), as well as coarse estimates of the cluster distance (column 9) and of the number of galaxies it contains (column 10). (© AAS. Reproduced with permission).

Most astronomical catalogues list one object per row. Columns typically begin with object identifiers (such as the object number in this given catalogue), followed by the celestial coordinates Right Ascension (similar to geographic longitude) and Declination (similar to geographic latitude, both at a certain epoch). These are typically followed by columns of specific measurements and their errors, such as the brightness (magnitude) in certain wavelength bands, the shape of objects, radial velocities and so on. Figure 1 shows an excerpt of George Abell’s (1958) catalogue of 2712 galaxy clusters in the northern and equatorial sky, based on his visual inspection of the 879 pairs of photographic plates^8^ of the Palomar Observatory Sky Survey. This catalogue was printed on 43 pages in an issue of the *Astrophysical Journal’s Supplement Series*.

**Figure 2.**
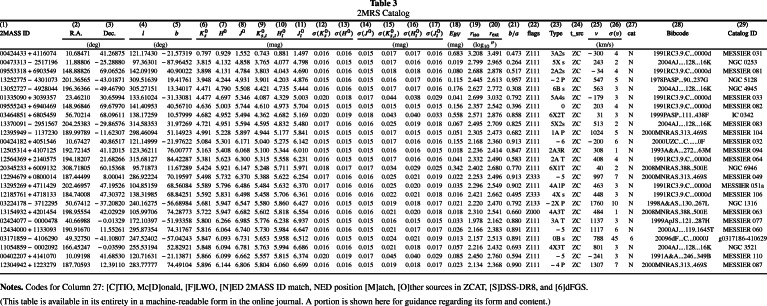
Structure of the 2MASS Redshift Survey (2MRS) catalogue (Huchra et al. 2012), which contains 44,599 nearby galaxies selected from the catalogue of 2MASS, a near-infrared all-sky survey, and supplemented with spectroscopic observations by John Huchra and his collaborators. For each object the columns list an identity number (column 1), celestial and galactic coordinates (columns 2 to 5), measured magnitudes in six infrared bands and their errors (columns 6 to 17), the galactic reddening (column 18; see Section 3.3), angular size and orientation (columns 19 and 21), flags (column 22; see Section 3.4), galaxy type (column 23), redshift and redshift uncertainty (columns 24 and 25), as well as additional information (columns 26 to 29). This is not a regular excerpt of the catalogue but, as noticed, a portion shown ‘for guidance regarding its format and content.’ (© AAS. Reproduced with permission).

Since the mid-1990s, large catalogues have been available electronically only, typically through data centres like the Centre des Données Astronomiques in Strasbourg (France) or the NASA Extragalactice Database (NED) at the Infrared Processing and Analysis Center at the California Institute of Technology in Pasadena, California (USA). Figure 2 shows an excerpt of the 2MASS Redshift Survey (2MRS) catalogue (Huchra et al. 2012), which contains information on 44,599 nearby galaxies selected from the catalogue of 2MASS, a near-infrared all-sky survey, as supplemented with spectroscopic observations. Yet larger catalogues are accessible only from relational databases through query languages like SQL. Examples are the catalogues of the Sloan Digital Sky Survey (SDSS), a digital photographic sky survey in five color bands supplemented by digital spectra (York et al. 2000), and the *Gaia* catalogue mentioned above.

The SDSS catalogues are among the most often used data products in astronomy. The SDSS collaboration has published approximately one new data release every year since 2002, drawing on a steadily increasing number of exposures, new detectors and improved data analysis procedures. Astronomical catalogues and data releases are fixed entities that are referenced like publications. Their version number always matters. It indicates the fixation of a specific state of data processing. As Alexander Szalay, a member of the SDSS collaboration, put it: ‘A [SDSS] data release is like a book: you can’t take it down – people use old editions’.^9^ All SDSS data releases since the 2002 SDSS Early Data Release are still available online.

### Which kind(s) of knowledge could a catalogue encode?

As with many other contemporary astronomical surveys, users of the SDSS can access the ‘raw’ data, such as digital photographic exposures, themselves and process these data ‘from scratch’ according to the specific requirements of their research project. Indeed, doing so is what David Hogg, a member of the SDSS collaboration, recommended, in principle, to users who want to exploit the information content of the SDSS’s photographic exposures maximally. But for most users the SDSS catalogues have an inestimable benefit, as Hogg explained in a talk at the Space Telescope Science Institute in Baltimore (Maryland, USA) in June 2011:

*Transcript 1*

The most important thing about catalogues is … they encode the collective knowledge of the people who make the data. So the Sloan catalog is the only place [where] we really encoded what we think the noise model of Sloan is … what we think the point-spread function is … what we think the data artifacts are. Because the catalog has been made sensitive to those things. So really … now somebody who works on the VO [Virtual Observatory] should be sweating here … because we shouldn't be passing forward these important metadata through the catalogue. But the reality is … we are … this is how we propagate these metadata! In fact … the VO has no total protocols for sending forward the noise model … the only way to understand the noise model is by looking how the catalogue is constructed.^10^

The SDSS catalogue’s metadata (‘data describing data’) contain basic general information on exposure times, column headings etc. only. Users can find more detailed, but not exhaustive, information on SDSS data processing in the data release papers accompanying every new release.

Hogg’s claim, that catalogues ‘encode the collective knowledge of the people who make the data’ may, in one sense, appear to be self-evident and uncontroversial. Of course, scientists ought to have made the best possible use of their knowledge in processing and analysing the data they make public. They are most familiar with the detectors and data analysis procedures used along the way. They collaborate in teams to make these data, and distribute their work, so ought they not have used their collective knowledge for everything they release? Yet, in another sense these remarks are profound: Would ‘collective knowledge’ – an elusive and controversial topic of philosophical debate – here become conceivable and consequential through its materialisation in a digital object? Much of the philosophical controversy revolves around whether the notions of ‘knowledge’ and ‘knowing’ can be meaningfully ascribed to collectives and not only to individuals.^11^ All of these views of Hogg’s claim are of interest for CSCW because of their relevance for mediating, instructing and coordinating cooperative work at a distance, and thus for theorizing coordinative artifacts.

A catalogue usually contains measurements, typically in the form of numbers. Measuring is a scientific practice that is inseparable from the organised ‘form of life’ (Wittgenstein 2009) of a discipline (Lynch 1991).  When made using digital photographic exposures, measurements are computed in specific ways using the numerical pixel values. For example, measuring the position of a star in a pixel image requires finding its center coordinates, whereas measuring its brightness requires adding the amount of its light recorded in several pixels. Both of these operations follow discipline-specific methods and protocols, including calibrations. They involve assessments of what is ‘good enough’ to complete a measurement, and to share it meaningfully. This work pertains to Thomas Kuhn’s (1961) view that scientific disciplines are communities bound by ‘concerted agreements on theories, measuring techniques, and characteristic modes of demonstration’ (Lynch 1991, p. 105, footnote 1). Measuring, and agreeing on the adequacy of measurements, is about sharing ‘common ground’ (Clark 1996).

Expert catalogue users know that its entries are the result of procedural work. They read other scientists’ measurements as the result of structured activities, ideally as the proper execution of protocols or rules of conduct. One may note the similarity to CSCW accounts of coordinative mechanisms that, likewise, conceive of coordinative artifacts to be tied to coordinative practices (Schmidt and Simone 1996; Schmidt 2011).

To know how to make, and how to use, measurements and catalogues properly is what Gilbert Ryle (1949, p. 133) would term a ‘capacity verb’ which ‘is used for signifying that the person described can bring things off, or get things right’. Ascribing knowledge to someone therefore presumes the witnessability of this person’s actions, or the products of these actions. Harold Garfinkel turned the problems of mutual understanding that Ryle and Wittgenstein considered into questions for empirical study. Responding to fellow sociologist Aaron Cicourel in a discussion on researching the acquisition of language skills, Garfinkel (in Hill and Crittenden 1968, p. 47) put it pithily:

‘know’ here has to do not with what one might have in mind in some secret place. It is not a case of your having to calm a respondent or seduce him in order for him really to tell you. Then you would be illuminated on what he had been hiding all along. Instead, ‘know’ consists really in a structure of activity. That is what the ‘know’ consists of.

Garfinkel here responds to attempts to locate knowledge in a place such as the mind or the brain. Conceiving of knowledge as a capacity or an ability (such as to exhibit a structure of activity) differs from the epistemological notion of knowledge as ‘justified true belief’. The latter has dominated philosophical discussions of collective knowledge. It presumes that knowledge ‘consists in possessing the right sort of belief in the right sort of propositions’ (Chang 2017, p. 103). In short, it is held to be propositional. Many of the philosophical troubles with ‘collective knowledge’ (a notion that I discuss further in Section 4.2) stem from disagreements about where to locate it. Yet for scientists, ‘[s]hared beliefs are much less common than shared practices’ (Netz 1999, p. 2; cf. also Rouse 2003; Chang 2017). If one considers knowledge as being embedded in systems of practice, then it is more appropriately described as an ability than as a belief. Doing so resonates with CSCW and STS accounts of scientific practice. It goes without saying that considering ‘knowing’ as a structure of activity emphasizes that it is witnessable and thus, in principle, accessible for empirical studies of cooperative work.

It is thus only in the way they contain higher-level data as processed from ‘raw’ data that catalogues could be said to encode collaboration members’ collective knowledge. One consequence of the unavoidably communal structure of scientific data analysis is that the same raw data can be processed into diverse forms of higher-level data specific to the evidential contexts of interest to members of different epistemic communities (Hoeppe 2014; Pinch 1985). For example, a digital astronomical exposure would be processed differently if one wanted to measure the extent of a diffuse gas nebula on the sky or the brightness of stars that appear point-like. Consequently, one would make separate catalogues for listing properties of gaseous nebulae and of stars. As higher-order data, the measurements in photographic exposures found in many catalogues relieve users from having to know the recording instrument and its characteristics, but this ‘externality’ comes at the price of measurements being specific to the evidential contexts of concern to catalogue makers.^12^

How data are released may reflect disciplinary, institutional or cultural commitments to open access. In astronomy, for example, successful data reuses are common (Pepe et al. 2014; Plant and Hanisch 2020). Most public (tax-financed) observatories release observational data at the end of periods of proprietary use by the scientists who had applied for these data (Hoeppe 2018; McCray 2017). Such data have been processed using standard routines in ways that are usually insufficient to count as evidence for any specific epistemic claim. Data releases may include data of low externality, such as uncalibrated photographic exposures of the sky, but their specific value is often marked by products of higher externality, such as catalogues, which, ideally, relieve users from the intricate work of calibrating detectors and further processing. This said, some uses may require a return to the observatory-processed, or even the unprocessed, ‘raw’ data.

### ‘A catalogue should be made algorithmically’

A point often made by astronomers is that object catalogues should be machine-generated, and not be made or edited by hand. That is, catalogue entries should be the unmodified output of processing data with computer code. Astronomers commonly say that catalogues that have been made in this way have been ‘made algorithmically’.^13^ This demand is consequential for articulation work and the temporality of projects. Astronomers David Hogg and Dustin Lang compare the usefulness of the Sloan Digital Sky Survey’s object catalogue, which is ‘machine made’ in this sense, with an influential ‘manually made’ catalogue, the Abell (1958) catalogue of galaxy clusters mentioned above (Section 2.1). The galaxy cluster Abell 2713 (pseudo-numeral), which was observed by the MUWAGS collaboration described below, is one of its entries. Hogg and Lang write:

[George Abell] spent thousands of hours poring over images of the sky; his Catalog communicated information he found in those images, so that other workers would not have to repeat the effort. This was at a time when you couldn’t just ‘send them the data and the code’. Indeed, Abell’s Catalog wasn’t constructed using code at all; there was no way to re-run the experiment, so the experiment had to be recorded and published in the form of the output catalog. (Hogg and Lang 2008, p. 1)

Equipped with computers and code, contemporary astronomers are not constrained in the way Abell was.

Calls for catalogues to be ‘made algorithmically’ are aspirations to replicability. Large data sets like the SDSS catalogues and the *Gaia* data releases can hardly be made otherwise. However, not editing the outputs of code often means to leave known artifacts in the catalogue. In astronomical image processing, for example, straylight or so-called cosmic ray hits leave traces in exposures, and these may be wrongly classified as objects or may corrupt the estimation of numerical parameters, such as when fitting the radial light profiles in galaxy images. Researchers may sort out problematic catalogue entries, for example, by visually inspecting the pixel images of suspect objects. They may also leave the catalogue as is, perhaps adding a column with quality flags. These are numerical or textual descriptors which alert users to potential issues with these entries. An astronomer told me that

*Transcript 2*

There are two kinds of attitude in astronomy. One attitude is: Release everything … release all your flags and tell the users how to use the flags. And the other attitude is: Clean the catalogue of anything that’s bad and release only a catalogue where every entry in the catalogue is good. And we [in the SDSS project] took this path [releasing unedited outputs and adding quality flags] but we could have easily taken this other path.

Making a machine-made catalogue is inevitably an iterative, sequential and temporal process. For instance, one cannot know initially which parameter settings of an object detection algorithm are best suited in working with data from a detector that one has not used before, or in a scientific setting that is unfamiliar. There is no catalogue in which all parameters of source detection codes are set optimally for its first run. Situated in the distributed work and divided labor of a research collaboration, the demand for working with algorithmically produced catalogues thus introduces a regress, a tension of temporality, as Chuck, a member of the MUWAGS collaboration, notices:

*Transcript 3*

Ideally a catalogue is machine-made and not the result of handwork. I mean … you have an algorithm and it takes you from the raw frames [photographic exposures] to the finished catalogue … a machine that can run on its own. Whenever you improve the algorithm you get a new machine-made product … upon seeing that here and there you got objects that you do not like … which may be erroneous. Where in your code is the reason why these [objects] haven’t been detected properly? You can change the code and then they come out just fine. But whenever you change your code it is not only that these objects change … but it’s always all objects that change … even when it’s only in the statistical noise. To improve the good objects while not changing the poor ones significantly is why you muddle through this again. The others’ numerical values may change … hopefully not on average … but they will change individually. And this is a discontinuity that you don’t want to get every three weeks or so … just because you have found something new. You do not want to make a new machine product again and again.

Assessing what Chuck calls ‘good objects’ and ‘poor ones’ is inevitably contextual and specific to a certain measurement or analysis. Using algorithms for making measurements of samples of objects is thus intertwined with ‘doing science’.

### ‘Before releasing the data you should have done science with it’

Besides the call for releasing only machine-made catalogues, a common sentiment among the astronomers I talked with is that one should only release higher-level data (such as catalogues) that have been used successfully for a scientific analysis. Says a senior astronomer:

*Transcript 4*

A good data release is something you can do science with. And if you’ve done science then those things that you’ve used should be your data release.

Another senior astronomer told me:

*Transcript 5*

My lesson from being in the survey business is that it’s only when you do science with the data that you learn how good they are … or if there are problems with the data. Many mistakes appear only then.

‘Doing science’ in these views means successfully using data of relatively high externality to address specific evidential contexts (Hoeppe 2014; Pinch 1985). Note that neither of the two astronomers claims that a scientific result needs to be replicated for data to be releasable. Their point rather seems to be that researchers ought to inspect data and analyses for their ‘believability’. Thus, in a training session for astronomy graduate students, a senior researcher, when asked how to tell if one’s data analysis code is good enough, was heard by a student as responding: ‘You get results that you think you can publish and people will believe you’.^14^ Much the same can be said about data reuses. This is a case of what Collins (2004) calls the ‘data analysts’ regress’: ‘The only way to tell if one’s data analysis is correct is to have it discover real effects, but the only way to find out if effects are real is to analyze data in a correct way’ (Collins 2004, p. 668).^15^ What Chuck noticed above (Transcript 3, Section 2.3) is a form of the data analysts’ regress. In distributed collaborative work this becomes a challenge for articulation work (Schmidt and Bannon 1992; Strauss 1988). Conversely, ‘doing science’ is one way of holding uses of algorithms accountable (cf. Shah 2018).

## Steps in the collaborative fixation of an astronomical catalogue

### The MUWAGS collaboration and its data

The MUWAGS (Multi-Wavelength Galaxy Survey) project was a collaboration of 30 astronomers from 10 countries, including tenured senior scientists, post-doctoral scholars and PhD students. The collaboration comprised three major sub-teams in charge of making three constituent data sets that were to be made consistent in a public data release: MAMBO, an optical survey of the galaxy cluster Abell 2713, previously made by core MUWAGS members, was supplemented with a mosaic of high-resolution images taken with the Hubble Space Telescope (HST) and an infrared map taken with the MIPS detector on board of the NASA satellite Spitzer. The aim of MUWAGS was to use the combined data set to study how the evolution of galaxies is affected by various environmental conditions in a cluster of galaxies. Given the mutually complementary expertises of its members, assembling the MUWAGS team was a synergizing effort in Bietz et al.’s (2010) terms. Its organization was similar to what Paine and Lee (2021), in their typology of coordinative entities, call a Principal Group, the chief difference being that the principal investigator of MUWAGS did not have central control over monetary and human resources, making the collaboration a more egalitarian arrangement oriented to consensual decision-making.

The MAMBO catalogue of A2713 was foundational to the MUWAGS data set. It had been prepared from a series of digital photographic exposures taken with a telescope in Chile through a set of broad- and medium-band filters transparent to light at specific wavelength ranges from the near ultraviolet to the near infrared. The combined set of calibrated images was used to detect objects algorithmically using the public code SExtractor (Source Extractor; Bertin and Arnouts 1996) and to measure their radiation fluxes through each filter. By fitting spectral models from a template library to the resulting measured spectral energy distributions, each detected object’s type (galaxy, quasar, star, white dwarf), photometric redshift (a measure of cosmic distance) and, for galaxies, stellar mass, was estimated and included in the catalogue.

The MAMBO images and catalogue were supplemented by a mosaic of digital photographic images of a cluster of galaxies taken with the Hubble Space Telescope (HST) Advanced Camera for Surveys (ACS) during 80 orbits around Earth. These high-resolution images were to be used for detailed studies of the morphologies of cluster galaxies and to measure the distortion of background sources due to gravitational lensing ascribed to the cluster’s mass distribution.

The HST ACS exposures were flatfielded (dividing science exposures by calibration frames to homogenize the sensitivity across the field), corrected for cosmic ray hits (deleting artifacts) and ‘drizzled’ (corrected for an image distortion due to the camera being off the optical axis) using standard software provided by the Space Telescope Science Institute. Subsequently, the resulting images were matched to a standard celestial coordinate system. Next, the SExtractor algorithm was used to produce an HST object catalogue. Thereafter, the completeness of this catalogue was assessed by inserting simulated objects into the digital images and estimating the rate at which they were detectable algorithmically. All galaxy light profiles were then fit with GalaxFit (pseudonym), a popular code for estimating galaxy morphologies quantitatively.

The third main contributing part of the MUWAGS data set besides the MAMBO and HST data was a mid-infrared map of the A2713 field, observed using the MIPS (Multiband Imaging Photometer for Spitzer) detector onboard the NASA satellite Spitzer. This map was used to generate a catalogue of the detected infrared sources.

Additional data sub-sets included X-ray, ultraviolet, and radio observations of the field. These extended the survey’s multi-wavelength coverage but were not included in the catalogue.

The final MUWAGS data release included the processed images, a catalogue of measured quantities of ca. 90,000 objects (mostly stars, galaxies, and quasars) as well as additional maps of weak gravitational lensing in the observed field of the sky. The catalogue was presented as a FITS (Flexible Image Transport System; Hanisch et al. 2001) table, a standard format in astronomy, and accompanied by a Data Release paper in a leading journal. The data were made available through the Space Telescope Science Institute’s archive, the Centre de Données Astronomiques de Strasbourg (France) and the collaboration’s website.^16^ Given the wide field observed (about the size of the full moon), many types of astronomical objects were contained in the images and the catalogue, making the data useful for various projects. Anticipated users were scientists working on galaxy cluster science, cosmological deep field studies, gravitational lensing and on improving photometric redshift techniques.

### Resolving dependencies between constituent data sub-sets

As MUWAGS team members strived to agree on a consistent catalogue, mutual dependencies between the three main constituent data sub-sets became occasional challenges for articulation work across the team. Preparing the catalogue gave progressively less and less room for re-processing the constituent data sets. This required assessments of the needs of catalogue users, in the team and beyond, and involved compromises and negotations of the catalogue’s contents and format.

In January 2007, a first comprehensive draft catalogue was circulated among team members. Called the J2007a catalogue, it was subsequently used for their science projects. This was a merger of the MAMBO catalogue with the SExtractor and GalaxFit outputs as run on the HST images. Measurements of infrared fluxes of detected objects and estimates of galaxy star formation rates were kept in a separate catalogue. Two month later, in March 2007, the J2007a catalogue was replaced by the J2007b catalogue. It contained improved algorithmic fits to the light profiles of detected galaxies (useful for the study of galaxy morphologies) and a revised definition of the ‘cluster sample’ – galaxies regarded as belonging to the cluster A2713. All these changes were considered unproblematic.

The detection of a mistake in the code that had been used to estimate the old (2003) photometric redshifts instigated the making of a third draft MAMBO/HST catalogue, the J2007c catalogue. It was circulated internally in late July 2007. Chuck explained in an email to the team:

I have removed a bug in the photo-z software, which should make only a difference for rather faint objects. I also enlarged the redshift window considered - again, matters only for low-S/N objects. Should not matter for R<22 objects really much.

Here ‘photo-z’ stands for photometric redshifts, ‘S/N’ for signal-to noise ratio, and ‘R < 22 objects’ designates a magnitude range that includes relatively bright objects in the field which were targets for most of the team’s research on the galaxy cluster.

Two days later, Eddie, the head of the infrared sub-project responded to the team in an email with the subject line ‘doh!’, expressing his exasperation that

My masses, SFRs [star formation rates], etc ALL USE THE OLD REDSHIFTS. I.e. NOT the J2007c redshifts. Worse than that, I do not have the code which does the masses, so the timescale to make new masses is LONG. This is a big deal -- it's a major SNAFU^17^ to change photoz version 1/2 way through a project (…)

Distance measures (like photometric redshifts) are needed to calculate absolute physical parameters such as galaxies’ stellar masses and star formation rates. Eddie continues his message wondering if revised masses and values could be ‘piggybacked’ somehow from other parameters listed in the catalogue.

Three days later Mallory, the team’s principal investigator, circulated her assessment of the situation in an email. She noticed that the revised photometric redshifts did not affect the agreement with team members’ published work much. This was at most ‘mildly irritating’. However, she agreed that the implications for the computed values of galaxy masses and star formations appeared to be serious. There was no straightforward way to ‘piggyback’ them. Furthermore, the completeness of the new catalogue would have to be re-assessed, implying considerable additional work for Chuck.

Following consultations with the team members involved, Mallory decided to return to the original 2003 redshifts, deeming the effort to fix the redshifts ‘worthwhile but ultimately (…) not enough of an improvement to justify the effort involved to bring everything else to the same system’. The J2007c catalogue was replaced by the J2007d catalogue.

After another six months, during which sub-teams continued their work using the J2007d catalogue, a revised catalogue (the J2008a catalogue) was circulated in February 2008. In an email to the team Mallory called it the first ‘all-singing all-dancing catalogue’. It was the first version that included infrared measurements, galaxy stellar masses and star formation rates. Thereafter only small changes were made until the public MUWAGS data release in November 2008.

This episode illustrates the dilemma pointed out in Section 2.3 (Transcript 3), that is, how dependencies between its constituent data sub-sets constrained the fixation of the MUWAGS catalogue. In this case it was not only that dependencies on the redshifts mattered for the decision to keep the old redshifts, but also that revising the star formation catalogue would delay further work toward the data release. There was progressively less and less room for re-processing constituent data sets.

### Guiding catalogue users by introducing and structuring redundancies

Skilfully prepared catalogues are designed to counter-act potential misuses. One way to do so is to introduce opportunities for instructing users beyond the prescriptive information provided in the data release publication.

A major step in moving toward the ‘all-singing all-dancing’ MUWAGS J2008a catalogue – the first version to merge the MAMBO, HST and MIPS data – was the assemblage of a proto catalogue, the J2007d catalogue. This table of 709 columns and ca. 90,000 rows (one for each objected detected in the HST images using SExtractor) collected the outputs of diverse computer code – positions, measured radiation fluxes, photometric redshifts, error estimates etc. – that members of the three core sub-teams had generated. It included duplicate information and various cross-checks, many comprehensible only to their makers. Different team members had used competing code, yielding contradictory numerical values. Some parameters, such as the celestial coordinates of detected objects, had been measured both in the high-resolution HST and the lower resolution, but deeper MAMBO exposures. Deciding which code’s output to choose was deferred in the early stages of team work, but now decisions had to be made. As Chuck explains:

*Transcript 6*

Before you use the data [for science] and produce catalogues … before you share these with anybody … you prepare tables that contain a host of descriptive information … many columns for each object. Ninety percent of those are completely irrelevant for making plots … that is … plots that will appear in an article or so. But they are relevant to get to the point at which you can trust a catalogue for preparing plots … scientific plots for papers. For what you do first … as part of your data reductions … is to make diagnostic plots for quality control … to check: ‘Have I done a mistake or do … at least … the data look good?’

The J2007d catalogue was too big to be shared meaningfully with any user beyond the team. At a collaboration meeting in November 2007 the number of columns for each detected object was to be reduced from 709 to about 200.

In the following I consider how the table size was reduced at this meeting, focusing on exchanges between Mallory, who moderated the discussion, and Chuck, the head of the optical sub-team and chief maker of the MAMBO catalogue. In order to illustrate the scientists’ understandings and their orientation to catalogue users I transcribe the discussion in relatively great detail. Doing so illustrates the contexts in which MUWAGS members agreed on what to leave in the catalogue, what to add and what to take out. I use elementary conventions of conversation analysis (Jefferson 2004).^18^

Early in this discussion, Chuck proposed to ‘de-redden’ the optical flux measurements of all objects. Astronomers agree that because of dust and gas in our Milky Way Galaxy light from all directions in the sky is ‘reddened’: light of shorter wavelengths is scattered more than that of longer wavelengths. How much the radiation fluxes of extragalactic objects are reddened depends on where they are in the sky relative to the band of the Milky Way. Objects behind the Milky Way are reddened most strongly. Galactic reddening can be estimated and subtracted from flux measurements. Since all objects in the MUWAGS field are in almost the same direction on the sky, the same galactic reddening correction was applied to the measured radiation fluxes of each object in the field. 
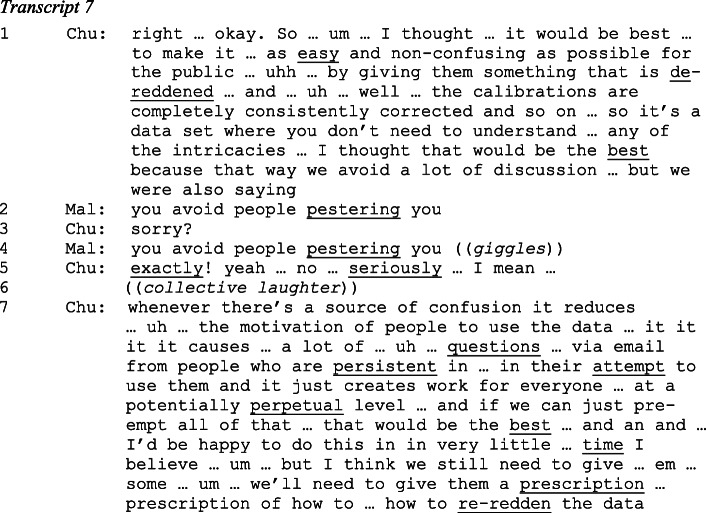


Chuck seeks to make uses of the catalogue ‘as easy and non-confusing as possible’ by listing de-reddened fluxes (line 1). Mallory’s suggestion that by doing so he would act in his own interest of not being ‘pestered’ by users (lines 2 and 4) is confirmed by Chuck who formulates this as a shared concern of catalogue makers and users (lines 5 and 7).

Considering the numerical catalogue entries in conjunction with matters that were practical otherwise, such as concerns over being ‘pestered’ by future catalogue users, was common in team discussions, not only at this meeting. Chuck and Mallory are concerned about the mistakes that catalogue users could make. The prospect of being ‘pestered’ by catalogue users suggests that interactions with users are possible, but, worried about its ‘potentially perpetual level’, Chuck rejects them in the pursuit of achieving the project’s closure. His desire not to be ‘pestered’ by catalogue users is curious in light of the opportunity of correcting misunderstandings that communications with users offer. It is well documented that data reusers often seek personal information from data makers when contextual information is insufficient (Carlson and Anderson 2007; Faniel and Jacobsen 2010; Rolland and Lee 2013; Zimmerman 2007). Yet, in a later conversation, Chuck told me that projects like SDSS and *Gaia* (see Section 2.1) were ‘public projects’ that were required to make public releases of processed data, and had the personal and financial resources to do so. By contrast, he considered MUWAGS as a ‘private project’ that was neither obliged nor sufficiently resourceful to do so. Seeking to pre-empt interactions with catalogue users was, for Chuck, an economical issue.^19^

In astronomical terms, de-reddening ‘de-localizes’ the data. It means to calculate the radiation fluxes one would measure when, hypothetically, observing the galaxy cluster from outside our galaxy. The de-reddened colours of extragalactic objects at different positions on the sky can be compared easily. Every practicing astronomer should be able to calculate de-reddened and re-reddened fluxes; this is taught in introductory laboratory courses for undergraduate students. In the data release paper team members could have simply mentioned the dust reddening correction that users would need to apply to obtain unreddened fluxes. Yet here – as elsewhere throughout the team’s discussions – there is the lingering expectation that data users would not read the data release paper carefully, thus being prone to make mistakes. Thus informed, the catalogue was designed to accommodate users’ projectable actions.

After agreeing that the optical fluxes would be de-reddened, Chuck suggests to leave a column with an uncorrected (‘un-de-reddened’) flux in the table: 
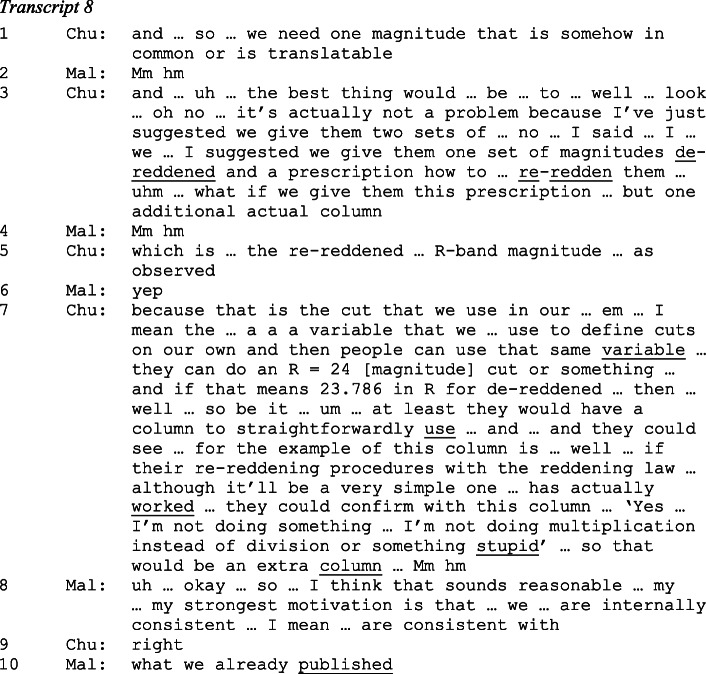


Chuck proposes (in lines 3 and 5) to provide an extra column in the catalogue that lists for each object a re-reddened magnitude (in the R band, the deepest image of the optical data set). This would give users interested in the uncorrected fluxes the opportunity to assess their calculations, which would be based on the re-reddening prescription as listed in the data release paper. Chuck makes this explicit when (in line 7), using reported direct speech, he takes an imagined user’s perspective. Mallory approves of including this – apparently redundant – extra column (lines 8 and 10), emphasizing her concern for the consistency of the data release (which was not affected by including this extra column), and the coherence with their own published work.

This was one of several exchanges in which team members debated introducing redundancies into the catalogue, and approved them. Note that with the extra column users are invited, or at least enabled, to perform a three-part sequence: (1) being instructed to re-redden galaxy magnitudes, (2) using these to calculate specific re-reddened magnitudes and (3) being afforded the opportunity to self-assess their results for one specific waveband (the R band). This sequence resembles a common feature of instructional sequences in classroom talk: a teacher asking a student a question, the student responding with an answer, followed by the teacher’s subsequent assessment of this response. This is the I-R-E (Initiation-Response-Evaluation) sequence, or Question with Known Answer (Lindwall et al. 2015; Macbeth 2003; Mehan 1979). Of course, catalogue makers and users are typically not co-present, users’ ‘response’ is in writing, and users self-evaluate their computation. Furthermore, students in the classroom are often allowed to speak only when called to respond to an utterance of the teacher. This is quite unlike the ‘lack of recipient accountability’ (Deppermann 2015) of written discourse. Only when it results in further writing, as scientific research ideally does, are the results of users’ ‘structures of activity’ (Garfinkel in Hill and Crittenden 1968, p. 47) revealed, and are then assessable retrospectively and made publicly accountable.

Taking the perspectice of imagined users, as Chuck did here, was a common feature of these discussions. That team members were able to do so is not surprising, given that they themselves often used other people’s catalogues and data sets. Chuck later explained to me in an interview:

*Transcript 9*

Every now and then I use other people’s data and want to do science with it … want to write papers [using it] … and there are factors that interest me as a catalogue user. I have caught myself thinking … ‘Gosh – now it’s getting too complicated with [using] this catalogue. What all do I have to know to use it properly and not come up with nonsensical interpretations … biased results?’ Perhaps the catalogue makers have provided lots of descriptive knowledge or whatever … but for me the situation may become uncertain as I don’t know how to use this knowledge and use the catalogue to transform it into the product that I wanted to have. And then I sit there and wonder: ‘Isn’t what I am actually looking for there somewhere on the web?’ And then I use it and that’s it. Or I let this paper go because the effort is getting too big.

Chuck describes himself as being an impatient reader of catalogue descriptions, arguably missing the guidance of the (numerical) catalogue entries themselves. A written description, it seems, can leave open too many ways of going astray.

### Guiding catalogue users by selectively deleting information and defining flags

Besides introducing redundancies in catalogue entries to allow users to apply cross-checks themselves, catalogue makers may delete information if their use is prone to mistakes, or to mark catalogue entries with flags – numerical or textual descriptors that alert users to restricted, potentially mistaken or contentious uses.

When the MUWAGS team met via teleconference in January 2008, the infrared-derived galaxy masses and star-formation rates, previously listed in a separate catalogue, had been merged with the MAMBO/HST catalogue. Now the usability of infrared-derived catalogue entries was in focus. During the call the following exchange unfolded between Mallory, Chuck and Eddie, the head of the infrared sub-team, who was in charge of the columns listing the star formation rates of galaxies: 
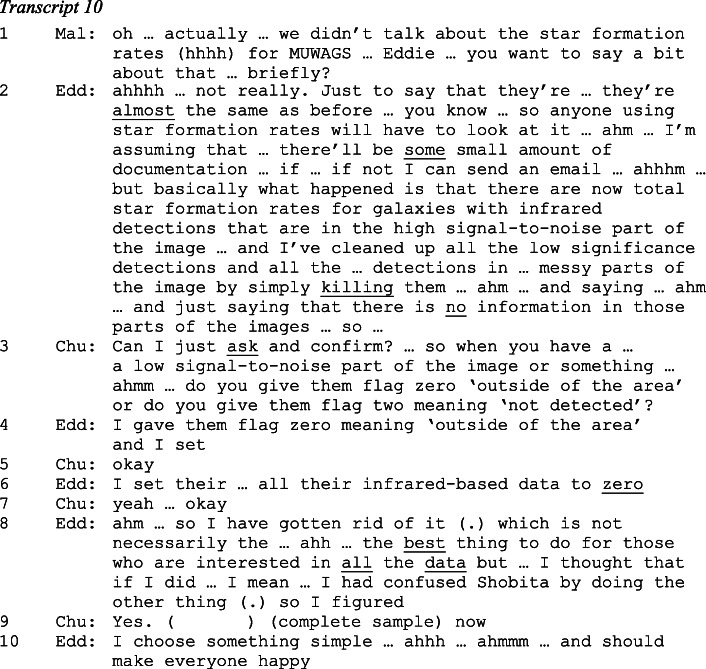


As in Transcripts 7 and 8, it is Mallory who allocates speakers’ turns at talk. Following her invitation for Eddie to update the team on his work on the star-formation rates, he declares, jokingly, not wanting to do so (in line 2), but then proceeds to report in detail on the changes he made to a previous version. The potentially contentious nature of his action – the ‘killing’ of information derived from ‘messy’ parts of the image – becomes noticeable through Chuck’s request for explanation (in line 3), which Eddie answers and continues to address (in lines 8 and 10) despite Chuck repeatedly acknowledging his understanding and acceptance (in lines 5, 7 and 9). Mallory remains silent throughout this exchange.

As the head of the MIPS infrared sub-team, Eddie was entitled to setting all entries for ‘messy parts of the [infrared] image’ to zero (line 6). What is a ‘messy’ part of an infrared image was not for the members of other sub-teams to judge. However, what Eddie describes as ‘killing’ has a moral connotation. Setting numerical *values* of the table to zero can be heard as an improper disregard for the *value* of these data, obtained as they were using the particularly precious observing time of a satellite telescope. Eddie acknowledges that deleting entries is not the best thing to do, but he emphasizes his orientation to avoid confusing catalogue users, maintaining that even Shobita, a MUWAGS team member, had been confused (in line 8). For Eddie, this concern for co-operation and intelligibility, among team members and beyond, overrides the effort to maximize the catalogue’s information content. Note that Shobita appears to have made visible to Eddie what he could not presume as an unquestioned background.

When Chuck and Eddie talk about ‘flags’ in this exchange, they refer to sample selection and quality flags.^20^ Quality flags are assigned manually to catalogue entries or are generated automatically by algorithms like SExtractor and GalaxFit. The MUWAGS collaboration defined quality flags for each of the constituent ‘sub-catalogues’. These flags were refined in the course of fixating the data release and writing the data release paper. At this stage in the discussion the MIPS catalogue had three sample selection flags: 0 (‘source not covered’, that is, not in the MIPS ‘footprint’ on the sky), 1 (‘source covered and detected’), and 2 (‘source covered, but not detected’ – that is, a flux density below the detection limit).

Reflecting on the formulation of quality flags, a collaboration member told me:

*Transcript 11*

You know … we insert a column for the dumb ones. This sounds arrogant … but what I mean is this: let’s pretend the public is dumb. And what we do is to tell them ‘Look – this is a column for you … and if you find this number there then just ignore this thing and use the rest only … before it’s getting too complicated … where too much can go wrong … where you have to know too much as a user … or where we would have to communicate too much too precisely … and we are not willing to make that effort’ … We try to simplify the situation. In that way you cannot use 100 percent of the power of the catalogue … but they can … let me just make up a number … it can be used to 80 or 90 percent by the dumbest possible user. At least nothing will go wrong. That is the point. Better leave opportunities untouched than to let users produce nonsense.

Thus conceived, the resort to flags is a shortcut to account for operations that are difficult to describe and prone to mistaken uses. Defining flags is also consequential for the temporality of the collaborative work of making the catalogue, particularly in dealing with the tension between producing catalogues algorithmically (Section 2.3) and the commitment to do science before releasing them (Section 2.4). Eddie’s assignment of flags marks the closure of work on the infrared data. These were not processed further.

### Using representational formats to deflect accountability

Where catalogue makers deem misuses of their work likely, they may introduce redundancies, delete information prone to misunderstandings, and introduce quality flags. They may also seek to deflect accountability by releasing data in a format that makes users visibly accountable for their (mis-)uses.

This came to matter in how the MUWAGS team released its information on weak gravitational lensing in the A2713 field. Potential ‘shareables’ of this analysis were the shear catalogue (a table of numerical measurements) and the kappa map, both representing the weak gravitational lensing shear – the deflection of the shapes of background sources in the field of the galaxy cluster. Christina, who prepared this map for her study of dark matter, was willing to share the map, but declined to share the shear catalogue. At a collaboration meeting she rehearses, in reported direct speech, an exchange she had with a user of her draft catalogue: 
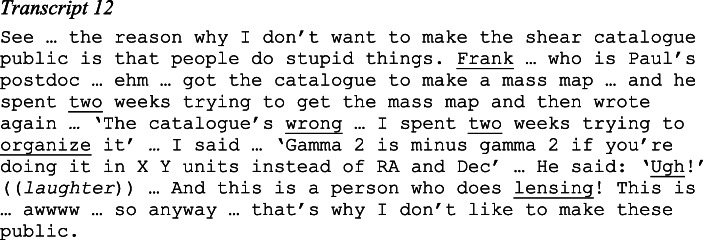


Here RA (Right Ascension) and Dec (Declination) are celestial coordinates, whereas X, Y units refer to positions in a pixel map. Frank, a user of her draft shear catalogue, can be heard as complaining about Christina sharing a faulty catalogue. She was alarmed that even an expert scientist working in the field (‘a person who does lensing’) had used the shear catalogue wrongly and appeared to blame her for it. If users had to make a shear catalogue from the ‘raw’ data themselves, they would have to be held accountable for their own mistakes. As well, if users retrieved numerical values from the kappa map – a representation of the shear catalogue in pixel format –, it would be them who would have to be blamed if these values turned out to be nonsensical. How users can, or cannot, use a data set is thus shaped by the representational formats in which it is presented (cf. Hoeppe 2019a). In this way notations and representational formats shape data (re-)uses and their accountabilities.

### Turning makers into users: testing the catalogue by trying to break it

A distributed team seeking to prepare a coherent data release faces specific challenges of articulation work (cf. Section 3.2), but the team’s internal diversity of expertises is also a resource for recognising how reusers external to the team may misunderstand released data. Team members’ joint engagement in testing illuminates the ‘object-ness’ of the catalogue. It assures the catalogue’s irreducibility to the knowledge of individual team members and illustrates the common membership of makers and anticipated users in an epistemic community.

In February 2008, following the circulation of the first MUWAGS catalogue that merged the MAMBO, HST and MIPS data, Mallory, the principal investigator, sent a message to the team, asking its members to break the catalogue: ‘Please try to break it. Please recreate your earlier plots and make sure everything still works as it should’. In doing so they were to inspect what had been achieved by combining the optical and infrared catalogues (Section 3.2), by reducing the table size (Section 3.3), by defining flags and by setting potentially confusing low infrared fluxes to zero (Section 3.4), among other things.

One afternoon I joined Antonio, a PhD student, in his effort to break the \alogue. He explained to me:

*Transcript 13*

What she wants to do is to compare the old catalogue with the new one that Chuck sent us and check if the numbers are more or less the same. Maybe there are a few differences … but they are not anything worrying … I would say the numbers are more or less the same. And then … when I have that feeling … that the numbers are more or less okay … I try to reproduce some of the old plots that were done with the old catalogue. Just in case. I’ll do it only with some of my plots … not all of them … because I do not feel like running all the code on the catalogue … but easier things … like [galaxy] colours … masses and things like that. So the mass … the star formation rate … that is what I work with mainly … have been slightly changed. That does not mean that it is not … that it is better or worse than in the past. That may be a difference … slightly … for the selections. But I am not going to change that for my paper … because everything is done … In terms of science nothing will be improved. Then I live with what I have.

Antonio began with uploading the catalogue and selecting an object sample by specifying the celestial coordinates (Right Ascension and Declination) of the cluster field, explaining to me that ‘it is a good thing first to check RA and Dec’. He then used the sample selection flags to count the number of objects in the field as pertaining to various combinations of sample selection flags (selecting ‘objects detected by MAMBO in the MUWAGS field’, ‘objects in the HST field detected by MIPS’ etc.). This done, Antonio proceeded by plotting the positions of objects in the field, assessing whether the distribution of objects looked reasonable. He explained to me that a reasonable distribution was one that showed the familiar pattern of the cluster galaxies with a relatively smooth random-like distribution of background sources. As such his work is informed by implicit assumptions of what the universe, and this part of it, looks like through the representational formats of the discipline (cf. Hoeppe 2014). Finding this plot acceptable, Antonio continued by recreating plots pertaining to his own project (on mergers of galaxies) that he had made using the draft catalogue. This included making color-mass diagrams for galaxies in different redshift bins. These plots looked good to him as well. He concluded this work after about two hours by stating, ‘I would say that in general the catalogue is right’ and communicated this assessment in an email to Mallory, the team’s principal investigator. Given his success of replicating the plots, Antonio is unwilling to revise his figures for the research paper that he was about to complete. He had achieved closure on his project and saw no need for a return to the reflexive correction of the data.

Two months later, in April 2008, at the last collaboration meeting before the data release, a ‘catalogue breaking session’ was held. During this session plots and science results were not re-considered, but the proper assignment of sample selection and quality flags was examined collaboratively. A few additional mistakes were found and the specifications of sample selection flags subsequently corrected. When no further possibilities of breaking it were discovered, the fixation of the catalogue was deemed complete. The catalogue was ‘frozen in’, as Mallory put it.

Attempts to ‘break’ the catalogue, and to ‘freeze’ it, figuratively assert its hardness, its materiality as an object. With each team member recreating their plots, they assessed the catalogue – now a singular digital object – in respect to the various evidential contexts that team members investigated. These included the distribution of cosmic dark matter, star formation and merger rates of galaxies, the properties of dwarf galaxies and the impact of different environments on galaxy evolution (cf. Section 3.1). The catalogue breaking session turned makers temporarily into users of their collective work. Their task was to approach the catalogue ‘from the outside’, as users would engage it. In so doing, team members’ limited mutual familiarity with the measurements of sub-teams to which they themselves did not belong became a resource for examining the coherence of the catalogue and for assuring its irreducibility to the knowledge of individual team members. Finding contradictory measurements or inconsistent quality flags and sample selection flags are examples of successful ‘catalogue breakings’. That team members took the perspective of data users in testing their own catalogue illustrates the presumed common membership of makers and potential users in an epistemic community.

## Discussion and conclusions

### Making an instructing data object for potentially unruly users

The MUWAGS catalogue was made to be acceptable to team members and coherent with the diverse data uses pertinent to their completed work. Making it was a negotiation involving compromises as well as efforts to guide proper uses and pre-empt mistaken uses by other scientists.

Although it would have been possible for members of the MUWAGS team to consult with potential users and design their data release accordingly, they did not do so. Yet, real and imagined users (and imagined uses) featured prominently in team discussions, where users’ actual and presumed actions and intentions were commonly represented by using reported direct speech (such as in Transcripts 8, 10 and 12). Imagined users were described as potentially ‘pestering’ the catalogue makers, as not reading instructions attentively (Section 3.3), and as being prone to make mistakes for which they could hold the team accountable (Section 3.6). In sum: viewed through their (imagined) actions, users were deemed (potentially) unruly – and so were (potential) uses of the released data.

A closer look reveals that these characterisations draw on team members’ self-reflection of their own conduct as professional astronomers. The designers that Woolgar (1991) studied had considered users as generic subjects that were to be configured, Sharrock and Anderson (1994) witnessed typifications of users, whereas in Martin et al.’s (2007) study users were ‘more proximal and real’. But for these astronomers, data makers and anticipated data users were agents who perform recognizably structured activities and who are scientists belonging to one epistemic community. MUWAGS team members were themselves users of other scientists’ data (cf. Transcript 9) and they drew on this experience as they completed the fixation of their catalogue. In a certain sense, team members became ethnographers of their own culture (Hoeppe 2020).

One could argue that these astronomers tried to remove what is indexical^21^ of their specific competence of using, *here* and *now*, *these* telescopes, *these* detectors and *these* algorithms and to make *these* data available for fellow scientists with the cultural competence of any – or many – astronomers who work with digital data, at least in this domain of studies of galaxy evolution, galaxy clusters and cosmic dark matter. However, it is one of ethnomethodology’s elementary, yet foundational, insights that one cannot get rid of the indexical altogether (Garfinkel 1967, pp. 4–7). Attempting to remove certain indexicals always leads to replacing them with others. In the present case one may say that these astronomers seeked to ‘re-indexicalize’ what was indexical of the local situation of data production with what is shared with, and accessible to, fellow extragalactic astronomers. As such they aimed to make their descriptions appropriate to a background of skills and knowledge that data makers and reusers share.

Since team members could not draw on communicational resources available for repair in face-to-face interaction, their challenge was to shape the data release so as to make it an object that instructs its users beyond the instructions provided by the data release paper. The episodes described in Section 3 reveal some methods for doing so. Among these were introducing and structuring redundancies that would help users to self-correct mistaken uses (Section 3.3), guiding users by selectively deleting data and defining sample selection flags (3.4), and deflecting accountability through making notational/representational choices (3.5). Yet other methods could certainly be identified, not the least ones pertaining to the presentation of digital data other than tabulations. To speak of methods here may seem exaggerated. After all, little of this was particularly noteworthy for these scientists themselves. Yet it is just their apparent ‘common sense’ that marks these ways of doing things as part of extragalactic astronomy’s form of life.

The use of redundancies may, at first, seem odd as a means for communication in science. Thus, Rogers (2014, p. 59) states that ‘[r] edundancies are common troublemakers in scientific communication’. However, this view is challenged by the diverse uses of redundancies in coding, data storage, cryptography and communications familiar since at least Shannon and Weaver (1964). An instructive case is the use of notation in mathematical writing, such as when formulating exercises. Knuth, Larrabee and Roberts (1987, p. 19) explain:

Exercises are some of the most difficult parts of a book to write. Since an exercise has very little context, ambiguity can be especially deadly; a bit of carefully chosen redundancy can be especially important.

Much as students who try to solve textbook problems, data reusers may lack knowledge and experience of the contexts that data makers have of detectors, algorithms and analysis procedures. In both contexts, redundancies are not merely duplicating information, but offer users a variety of sequential engagements to assess their understanding.

That repetitions of utterances in talk-in-interaction are not meaningless is a central lesson of pragmatic understandings of language, including those aligned with ethnomethodology and conversation analysis. For the latter, actions and utterances are unavoidably indexical, and, as such, the same word can have different meanings when repeated or found in different sequential positions (Garfinkel 2008). In Sections 3.3, I have noticed team members’ aspiration to design redundancies that would instigate three-part sequences to instruct catalogue users. These resemble I-R-E (Initiation-Response-Evaluation) sequences – or Questions with Known Answers – characteristic of classroom repair (Lindwall et al. 2015; Macbeth 2003; Mehan 1979). Their triadic structure builds on what Sacks et al. (1974, p. 728–729) refer to as a ‘proof procedure’ for the analysis of turns in conversation. Sacks et al. argued that the talk of a speaker in response to a previous utterance displays this speaker’s understanding of the utterance to co-participants of a conversation. In this sense the participants to a conversation are the ‘first analysts on the scene’ (Macbeth 2003, p. 241).

Schegloff (1992) argued that, in talk-in-interaction, the speaker’s repair after the next turn of the respondent is the ‘last stand’ of intersubjectivity in conversation (cf. Macbeth 2011, p. 440). In sharing scientific data with potential reusers, scientists cannot hope to achieve intersubjectivity in this sense. Much rather, as pointed out in Section 3.3, exchanges with users were, by members of the MUWAGS team at least at times, actively disencouraged in the attempt to achieve closure. However, a catalogue can instruct its users through well-designed redundancies and by offering diverse cross-checks,^22^ even when instructional three-part sequences remain incomplete in practice and the achievement of mutual understanding is, in the end, not guaranteed (Wittgenstein 2009: para. 145).

The intended instigation of three-part sequences in catalogue uses can be contrasted meaningfully with accounts that emphasize the chasm between oral and written communication. For instance, Krämer (2015, p. 23) argues that ‘[t]ransmission is precisely not dialogical: the goal of technical communication is emission or dissemination, not dialogue. We can thus clearly distinguish between the personal principle of understanding and the postal principle of transmission’. In instructing their users through the catalogue itself, the MUWAGS team seeked to go beyond this distinction and afford users with opportunities to self-correct their uses. Suchman’s (2007, p. 192) wariness of the limits of inscribing users and uses into design is bound to apply to scientific data releases and other instructing data objects nevertheless.

Note the importance of document formatting in this work. As with coordinative artifacts, lists and tables afford specific uses and help reducing ambiguity (cf. Goody 1977; Schmidt and Wagner 2004). Their spatial structure, order and notational characteristics afford diverse uses which are ‘foundational for coordinating activity distributed in time and space’ (Bowker and Star 1999, p. 138). But other formats also afford these functions, including tables, documents of various kinds (Anderson and Sharrock 2018; Smith 2001) and digital images (Hoeppe 2019b). The formats can be regarded as examples of what Goodwin (2013, 2018) calls a substrate, ‘an immediately present semiotic landscape with quite diverse resources that has been given its current shape through the transformative sequences of action that culminate, at this moment, in the current action’ (Goodwin 2013, p. 11). That ‘the substrate, and the resources it provides, makes possible specific forms of subsequent action’ (Goodwin 2013, p. 11) is what members of the MUWAGS team arguably seeked to utilize by operationalizing potential uses for users who are not co-present, do not belong to the same organization, and may not even live at the same time. With Goodwin’s understanding of the co-operation (with the hyphen) afforded by substrates, forms of Computer Supported Co-Operative Work (also with the hyphen) beyond a focus on group work become conceivable for CSCW at the granularity of its written and computational artifacts (cf. also Schmidt and Wagner 2004).

### (How) does a catalogue encode ‘collective knowledge’?

Inspired by astronomer David Hogg’s claim that astronomical catalogues ‘encode the collective knowledge of the people who make the data’ (Transcript 1, Section 2.1), I wondered if ‘collective knowledge’ here becomes conceivable and consequential through its materialisation in a digital artifact. In the following I give a very brief overview of recent philosophical work on ‘collective knowledge’ and assess its relevance for interpreting my account of Section 3.^23^

Philosophical accounts distinguish summative views of collective knowledge from those that insist on its irreducibly collective nature. Both are propositional, that is, they presume that knowledge ‘consists in possessing the right sort of belief in the right sort of propositions’ (Chang 2017, p. 103).^24^ The summative view asserts that ‘a collective knows p iff [if and only if] each member knows p’ (de Ridder 2014, p. 38). As de Ridder and others have noticed, collective knowledge, thus conceived, is reducible to the knowledge of individuals and so there is nothing distinctively collective about it. Views that consider collective knowledge as irreducible to that of individuals are of greater interest. For example, a committee could arrive at a certain position without each, or any, individual member subscribing to it (Wray 2017; cf. Beatty 2006). If one adopts the commonly held philosophical notion of knowledge as ‘justified true belief’ – as all of these accounts do, or, at least set, set out from – one may regard human collectivities as ‘epistemic subjects’ that can hold beliefs collectively. This is what Margaret Gilbert (2000) argues for in her ‘plural subject theory’. Brad Wray (2007, 2017) objects to Gilbert’s view by arguing that ascribing shared *acceptance* to a collective is more plausible than positing a shared *belief*. He formulates a novel definition of collective knowledge as ‘justified true acceptance’. In either case, the collectivity of knowers is delineated by those who believe or accept a claim. Whereas Gilbert extends collective beliefs to disciplines and adherents of Kuhnian paradigms, Wray (2007) confines collective acceptance to research teams and committees, arguing that only these have specifiable decision procedures. Probing another part of the definition of knowledge as ‘justified true belief’, de Ridder (2014) suggests to attend to the unavoidably collective justification of knowledge in science. It is, for example, beyond any individual scientist’s capacity to justify or evaluate findings of elaborate experiments. Questions of collective knowledge then turn into questions of justification, and ultimately into questions of trust (Hardwig 1991, cf. also Wagenknecht 2016: Chapter 8).^25^

It is possible to interpret the fixation of the MUWAGS catalogue in light of these considerations. If one sets out with conceiving of knowledge as ‘justified true belief’, both the replacement of ‘belief’ by ‘acceptance’ (Wray 2007, 2017) as well as the unavoidably collective justification (de Ridder 2014) of its contents are recognizable. The collective authorship of the data release paper signals the collective acceptance of the data set that it describes. The episodes presented in Sections 3.3 to 3.6 illustrate how the justification of catalogue entries and quality flags relied on members of sub-teams. That the final catalogue became irreducible to individuals’ knowledge is illustrated by the diverse evidential contexts engaged by team members. The episodes of Section 3 also illustrate the team’s decision procedures, involving the guidance and authority of the principal investigator (Section 3.2), as well as the reliance on diverse experts in the design of catalogue entries.

With the materials presented in Section 3, this discussion of collective knowledge can be enriched in two ways. The first is to take the materiality and mediality of writing into account and point out how it matters for the fixation of data sets that are irreducible to the work of individual team members. The second is to move from propositional notions of knowledge to conceive of knowing as a ‘capacity’ (Ryle) or ‘structure of activity’ (Garfinkel). I argue that doing so is essential for making sense of the MUWAGS catalogue as an instructing data object.

The philosophical literature reviewed above touches on the role of the materiality of writing for its conceptions of collective knowledge at most implicitly, such as when Wray (2017) discusses Beatty’s (2006) example of how an expert committee arrived at an assessment of radiation safety. This involved the use of documents. Discussions of collective authorship imply the use of documents as well (e.g. Hardwig 1991; Huebner et al. 2017).

MUWAGS members’ attempts to ‘break’ their catalogue figuratively asserts their understanding of the catalogue’s hardness as well as its integrity and materiality as an object (Section 3.6). Alluding to the catalogue as a ‘breakable’ object is reminiscent of descriptions of software as breakable or brittle in its use (Dourish 2017; Hoeppe 2014; Rooksby et al. 2009; Spencer 2015; Whittaker 2002). But what constitutes the perceived hardness or materiality of the MUWAGS catalogue? The discovery of inconsistencies of catalogue entries, including of sample selection flags, were considered instances of ‘catalogue breaking’ that had to be fixed. This done, the completed catalogue was deemed ‘hard’ because it enabled diverse uses coherently, was logically consistent and accountable to the objects of scientific discourse. This implied understanding of ‘hardness’ may remind one of the hardness of logical necessity that Wittgenstein (2009, para. 97) pointed to. Attempts to break the catalogue were a sort of usability trial (cf. Rooksby et al. 2009) that was conducted within the team. The internal diversity of the uses of team members came to matter in confirming the ‘hardness’, and successful fixation, of the catalogue.

Along with the team’s collaborative process of merging the outputs of team members’ code (Section 3.2), which pertained to addressing diverse epistemic contexts, their conduct of usability trials made the catalogue irreducible to the work and knowledge of any individual team member. Their successful use of the catalogue for addressing diverse epistemic contexts confirmed the catalogue as an agreed-upon common ground for this work and signalled its robustness to users.^26^

My second point is to support a move away from propositional accounts of knowledge as ‘justified true belief’ or ‘justified true acceptance’ toward ones that conceive of knowing as a ‘capacity’ (Ryle) or a ‘structure of activity’ (Garfinkel). As Ryle (1949, p. 133–134) put it, whereas ‘know’ is a ‘capacity verb … that is used for signifying that the person described can bring things off, or get things right’, ‘believe’ is a ‘tendency verb and one which does not connote that anything is brought off or got right.’ As argued in Section 2.2, there are good reasons to consider knowing as an ability when considering scientific practice. Garfinkel’s understanding of knowing as a ‘structure of activity’ (in Hill and Crittenden 1968, p. 47; cf. Section 2.2) puts this view to use in ethnomethodology. Note that, in holding this view, Garfinkel explicitly counters arguments that locate knowledge in the brain – arguably the root cause of the philosophical controversy on collective knowledge. Garfinkel insists that the ‘appropriate image of a common understanding is therefore an operation rather than a common intersection of overlapping sets’ (Garfinkel 1967, pp. 30–31). He is concerned with ‘a *procedural* sense of common or shared, a set of practices by which actions and stances could be predicated on and displayed as oriented to “knowledge held in common” – knowledge that might thereby be reconfirmed, modified, and expanded’ (Schegloff 1991, p. 152; emphasis in original).

A catalogue cannot coerce its users, but one that has been made well will be a resource for their actions. Yet it can instruct only users who are familiar with the settings in which it is usable (cf. Harper 1998, p. 38). Its makers can try to anticipate future uses, and thereby seek to shape and guide the structure of users’ activities. In the episodes of Section 3, MUWAGS members consistently oriented to what users could be expected to *do* with it. Formatted as a table, the catalogue invites structured uses by those who are broadly familiar with the procedures used to generate its entries, such as finding and using a column with de-reddened galaxy magnitudes (Section 3.3) to check one’s own uses of the magnitudes listed in other columns. It is thus with an understanding of knowledge not as exclusively propositional (such as the belief in propositions), but – in accordance with Ryle, Wittgenstein and Garfinkel – as embedded in witnessable activities, or the products of these activities, that Hogg’s claim (Transcript 1) is meaningful from a philosophical and ethnomethodological perspective.

### ‘Using algorithms’ versus ‘doing science’ in the fixation of a catalogue

In Section 2 I identified two widely asserted desiderata as to what digital scientific data releases should be like. First, they ought to be made ‘algorithmically’ (Section 2.3) and, secondly, prior to their publication, data should have been used successfully for scientific analyses (Section 2.4). The former is a demand stemming from aspirations to replicability, the latter is a demand for data to be usable as evidence pertaining to evidential contexts addressed by team members. Considered in the temporal and sequential work of fixating a data set these two desiderata can be seen as conflicting. They are a challenge for a collaboration’s articulation work.

The strict reproducibility of machine-made data sets has led scientists like Hogg and Lang (2008) to question the uses of catalogues altogether. Hogg and Lang speculate that the Sloan Digital Sky Survey (SDSS) collaboration (cf. Section 2.3) need not have produced a catalogue but could have released its calibrated digital images and its code, so that users could make object catalogues themselves. Thanks to the digitality of data and code, such catalogues would be exactly reproducible in principle. This is unlike Abell (1958) preparing his catalogue of galaxy clusters. Working manually in the 1950s, he could not automatize the visual assessments that he entered into his catalogue.

By contrast, the desideratum of having done science with data before its public release pertains to the suitability of data to serve as evidence for addressing certain evidential contexts (Section 2.4). This is not about reproducing specific numerical results exactly, but about producing results that can stand up to peer assessments. Doing so pertains to what discursive objects are like, what counts as sameness and difference, and how to make proper distinctions. These are assessments that members make qua their membership in an expert community. Thus conceived, a representation of natural order is tied to the (re-)production of social order.

The episode described in Section 3.2 and Chuck’s assessment in Transcript 3 (Section 2.3) illustrate how pursuing these two desiderata can be in tension in the fixation of a catalogue. Measuring object properties in digital images algorithmically and doing science are intertwined: a change to the algorithm may necessitate the re-assessment of a scientific result which, in turn, may require an adjustment of the algorithm (Hoeppe 2014). This is a sort of (ethnomethodological) reflexivity, with its temporality and delimitation unfolding in an organizational context. For a distributed research collaboration like MUWAGS that consists of sub-teams its challenges are aggravated. What is required is an interweaving of technical and organizational work (cf. Button and Sharrock 1998, p. 97). Assessments of what is ‘good enough’ under the constraints of time and circumstance are required to break what is, in principle, an infinite regress. Defining and introducing quality flags is one way to break it, as described in Section 3.4.

Note that there only is a tension between ‘producing catalogues algorithmically’ and ‘releasing data only after having doing science with it’ when considering data pertaining to higher externality (cf. Section 2.2). If ‘doing science’ means to address specific evidential contexts there is no ‘doing science’ with data of low externality. After all, unprocessed data cannot refer to the topical and disciplinary content of a specific epistemic claim directly. Unprocessed data, too, are contingent on social, technical and contextual decisions which have entered the construction of detectors and instruments as well as their uses (Latour and Woolgar 1986). But only data pertaining to higher externality address specific epistemic claims and disciplinary contexts. Only there do we find these data to agree or disagree with theoretical expectations about the objects of disciplinary discourse. It is in this sense that social and natural orders manifest themselves together in the fixation of a data set. This entanglement becomes itself a resource for the use of these data (Hoeppe 2014). In this way the discourse of a science becomes a resource for the collaborative fixation of a data set beyond the philosophical notion of the theory-ladenness of observation (Hanson 1958). The latter does not capture the essential and unavoidable sociality of this work. Although removed from concerns of everyday life and its orderliness we nevertheless find Garfinkel’s (1967, p. vii) observation supported, that ‘in doing sociology, lay and professional, every reference to the “real world,” even where the reference is to physical or biological events, is a reference to the organized activities of everyday life.’ In this sense, and perhaps without noticing it, the scientists of the MUWAGS team were doing sociology all along.

## Footnotes

1 What counts as ‘large data sets’ and ‘large teams’ is subject to changing views. Nothing in this paper depends on a definition of either. I specify uses of ‘large’ numbers in the case presented in Section 3.

2 Distinguishing ‘data reuse’ from ‘data use’ unambiguously is difficult (van de Sandt et al. 2019). In this article I focus on the preparation of measurements, a form of higher-level data, for the (re-)use by scientists who did not make them.

3 I am grateful to an anonymous reviewer for this formulation.

4 Note that the sequentiality of written instructions and protocols is distinct from the sequentiality in ethnomethodological and conversation analytical accounts of the local production and maintenance of order and mutual understanding.

5 My use of ‘fixation’ is inspired by Peirce (1992), who uses it to emphasize the processual, methodical and context-dependent arrival at a shared belief.

6 Using recordings of naturalistic settings along with interviews is a mix of methods. I make the distinction between these materials visible by transcribing them in different fonts. In this text, interviews are mostly participants’ comments on interactions that I witnessed previously in naturally occurring conversations.

7 http://vizier.u-strasbg.fr/viz-bin/VizieR. Accessed 11 June 2021.

8 For the Palomar Observatory Sky Survey each field ‘was photographed twice, once on a [blue-sensitive Kodak] Eastman type O emulsion and once on a type E emulsion through a red Plexiglass filter’ (Abell 1958, p. 121).

9 University of Heidelberg Astronomical Colloquium, Heidelberg (Germany), May 22, 2007.

10 http://realserver4.stsci.edu/t/data/2011/06/2642/DavidHogg061611.mp4 at min 11 (retrieved 5 Feb 2013). Noise models (which quantify the effects of the length of exposure time and detector properties on the image quality and, consequently, parameters derived from them), point-spread functions (which quantify the blurrying of starlight due to atmospheric turbulence and telescope and camera optics) and image artifacts (present in any astronomical exposure) are specific to the processing of digital exposures, such as those generated by the CCDs (charged-coupled devices) of the SDSS telescope at Apache Point in New Mexico (USA). See Hogg and Lang (2008) for an elaboration of this view and its consequences.

11 I review these views in Section 4.2 below. Concepts of knowledge are so diverse and have such long histories that it seems impossible to come up with a single meaningful definition, vindicating Friedrich Nietzsche’s (2014, p. 268) assessment that ‘only that which has no history is definable’. In this article I focus on the empirical study of documents and witnessable practices.

12 This does not mean that measurements are necessarily specific to a Kuhnian paradigm (cf. Chang 2011; Hoeppe 2019a).

13 As such this is a members’ term. Considered from a CSCW and STS analyst’s viewpoint, uses of algorithms are unavoidably situated in the material, relational and temporal work of making and using software (cf. Cohn 2019). This comes to matter, for example, in Section 3.2 (below), where Eddie’s lack of access to code that a former PhD student of his research group had developed complicates merging the constituent visible-light and mid-infrared catalogues.

14 http://jessresearch.blogspot.ca/2010/06/coding-wisdom-from-david-hogg.html. Accessed 25 December 2013.

15 In the astronomical work that I witnessed this regress was commonly resolved through what philosophers of science call robustness reasoning, which dwells on the convergence of ‘multiple means of determination’ (Wimsatt 2012, p. 61) toward a result (Hoeppe 2019a).

16 See https://archive.stsci.edu/ and http://cdsweb.u-strasbg.fr/. Accessed 1 June 2021.

17 ‘Situation Normal: All Fucked Up’, cf. https://en.wikipedia.org/wiki/SNAFU. Accessed 2 April 2020.

18 In the Jefferson (2004) transcription scheme underlining indicates stress, left side square brackets indicate where overlapping talk begins, empty parentheses indicate talk too obscure to transcribe, single round brackets indicate dubiously heard words, and double round brackets add the transcriber’s commentary. I deviate from the Jefferson scheme by transcribing pauses with dots, using quotation marks to mark what I heard as reported speech, and using full stops where I heard the end of sentences.

19 Not being ‘pestered’ by catalogue users is, for Chuck, an aspiration and not necessarily the achieved outcome of his work. He told me that, in fact, he does at times interact with users of his published data, instructing them on using the catalogue properly.

20 Table 9 of Stoughton et al. (2002) provides a comprehensive list of quality flags adoped by the Sloan Digital Sky Survey for its early catalogues.

21 In Garfinkel’s understanding, indexicality refers to the unavoidable context-dependence of actions (cf. Garfinkel 1967, pp. 4–7).

22 In the case of the MUWAGS catalogue, these included the numbers of objects in specifically selected object samples, as listed in the data release paper and the README file accompanying the data files.

23 More radical than the philosophical views of collective knowledge that I review is Karin Knorr-Cetina’s (1999) claim that an entire experiment in high-energy physics is a knowing agent. Since she focuses on symbolic classifications whereas I consider the collaborative production of a document it is difficult to relate Knorr-Cetina’s claim to my account. Another approach to consider the fixation of scientific knowledge in an artifact is Baird’s (2004) study of scientific instruments.

24 Note that a propositional understanding of knowledge is also shared by sociologists like Barry Barnes and David Bloor (1982), who ‘refer to any collectively accepted system of belief as “knowledge”’ (1982, p. 22, footnote 5).

25 This is an incomplete summary of the debate. See the references in Dragos (2019) for further contributions.

26 See Hoeppe (2019a: Section 3.3) on the robustness of data.

## References

[CR1] Abell, George O. (1958). The Distribution of Rich Clusters of Galaxies. *Astrophysical Journal Supplements (ApJS)*, vol. 3, no. 2, pp. 211–288.

[CR2] Akrich, Madeleine (1992). The De-Scription of Technical Objects. In *Shaping Technology / Building Society*. Wiebe Bijker; and John Law (eds.). Cambridge, Mass.: MIT Press, pp. 202-225.

[CR3] Anderson, Robert J.; and Wes W. Sharrock (2018). *Action at a Distance: Studies in the Practicalities of Executive Management*. Routledge.

[CR4] Baird, Davis (2004). *Thing Knowledge: A Philosophy of Scientific Instruments*. University of California Press.

[CR5] Barnes, Barry; and David Bloor (1982). Relativism, Rationalism and the Sociology of Knowledge. In M. Hollis; and S. Lukes (eds.). *Rationality and Relativism*. MIT Press, pp. 21 – 47.

[CR6] Beatty, John (2006). Masking Disagreements Among Experts. *Episteme*, vol. 3, no. 1-2, pp. 52-67.

[CR7] Bertin, E.; and S. Arnouts (1996). SExtractor: Software for Source Extraction. *Astronomy and Astrophysics Supplement Series*, vol. 117, no. 2, pp. 393 – 404.

[CR8] Bietz, Matthew J.; Eric P.S. Baumer; and Charlotte P. Lee (2010). Synergizing in Cyberinfrastructure Development. *Computer Supported Cooperative Work (CSCW)*, vol. 19, no. 3–4, pp. 245–281.

[CR9] Bietz, Matthew J.; and Charlotte P. Lee (2009). Collaboration in Metagenomics: Sequence Databases and the Organization of Scientific Work. In E. Balka, L. Ciolfi, C. Simone, H. Tellioğlu and I. Wagner (eds.). *ECSCW 2009: Proceedings of the 11th European Conference on Computer Supported Cooperative Work*, Vienna, Austria. Springer-Verlag. pp. 243–262.

[CR10] Birnholtz, Jeremy P., and Matthew J. Bietz (2003). Data at Work: Supporting Sharing in Science and Engineering. In: M. Pendergast (ed.): *GROUP 2003. Proceedings of the 2003 International ACM SIGGROUP Conference on Supporting Group Work. *New York: ACM Press; 2003, pp. 339–348.

[CR11] Blackler, Alethea L.; Rafael Gomez; Vesna Popovic; and M. Helen Thompson (2016). Life Is Too Short to RTFM: How Users Relate to Documentation and Excess Features in Consumer Products. *Interacting with Computers*, vol. 28, no. 1, pp. 27-46.

[CR12] Borgman, Christine L. (2015). *Big Data, Little Data, No Data: Scholarship in the Networked World*. Cambridge, Mass.: MIT Press.

[CR13] Bowker, Geoffrey C. (2005). *Memory Practices in the Sciences*. Cambridge, Mass.: MIT Press.

[CR14] Bowker, Geoffrey C.; and Susan Leigh Star (1999). *Sorting Things Out: Classification and Its Consequences*. Cambridge, Mass.: MIT Press.

[CR15] Brewer, Peter (2017). ‘Do You Expect Me to Just Give Away My Data?’ https://eos.org/editors-vox/do-you-expect-me-to-just-give-away-my-data?utm_source=eos&utm_medium=email&utm_campaign=EosBuzz091517 (accessed 7 October 2017).

[CR16] Button, Graham; and Wes Sharrock (1998). The Organizational Accountability of Technological Work. *Social Studies of Science*, vol. 28, no. 1, pp. 73-102.

[CR17] Carlson, S.; and B. Anderson (2007). What are Data? The Many Kinds of Data and Their Implications for Data Re-use. *Journal of Computer-Mediated Communication*, vol. 12, no. 2, pp. 635–651.

[CR18] Chang, Hasok (2011). The Persistence of Epistemic Objects Through Scientific Change. *Erkenntnis*, vol. 75, no. 3, pp. 413 – 429.

[CR19] Chang, Hasok (2017). Operational Coherence as the Source of Truth. *Proceedings of the Aristotelian Society*, vol. 67, part 2, pp. 103 – 122.

[CR20] Chin, G.; and Lansing, C.S. (2004). Capturing and Supporting Contexts for Scientific Data Sharing via the Biological Sciences Collaboratory. In *CSCW ‘04 Proceedings of the 2004 ACM Conference on Computer Supported Cooperative Work*. New York: ACM Press, pp. 409–418.

[CR21] Clark, H.H. (1996). *Using Language*. Cambridge, MA: MIT Press.

[CR22] Cohn, Marisa Leavitt (2019). Keeping Software Present: Software as Object for a Timely STS Studies of the Digital. In: Vertesi, Janet and David Ribes (eds.). *digitalSTS: A Field Guide for Science & Technology Studies*. Princeton, NJ: Princeton University Press, pp. 423 – 446.

[CR23] Collins, Harry M. (2004). *Gravity’s Shadow: The Search for Gravitational Waves*. Chicago, Ill.: University of Chicago Press.

[CR24] Curty, R.G.; K. Crowston; A. Specht; B.W. Grant; E.D. Dalton (2017). Attitudes and Norms Affecting Scientists' Data Reuse. *PLoS ONE*, vol. 12, no. 12: e0189288. 10.1371/journal.pone.0189288 PMC5744933 29281658

[CR25] Deppermann, Arnulf (2015). Retrospection and Understanding in Interaction. In *Temporality in Interaction*. A. Deppermann; and S. Günthner (eds.). Amsterdam and Philadelphia: John Benjamins, pp. 57-94.

[CR26] de Ridder, J. (2014). Epistemic Dependence and Collective Scientific Knowledge. *Synthese*, vol. 191, no 1, pp. 37-53.

[CR27] Djorgovski, S. George; Ashish Mahabal; Andrew Drake; Matthew Graham; and Ciro Donalek (2013). Sky Surveys. In: *Planets, Stars and Stellar Systems*. Edited by Terry D. Oswalt and Howard E. Bond. Dordrecht: Springer pp. 223-281.

[CR28] Dourish, Paul (2017). *The Stuff of Bits: An Essay on the Materialities of Information*. Cambridge, Mass.: MIT Press.

[CR29] Dragos, Chris (2019). Groups Can Know How. *American Philosophical Quarterly*, vol. 56, no. 3, pp. 265-276.

[CR30] Edwards, Paul N. (2010). *A Vast Machine: Computer Models, Climate Data, and the Politics of Global Warming*. Cambridge, Mass.: MIT Press.

[CR31] Edwards, Paul N.; Matthew S. Mayernik; Archer L. Batcheller; Geoffrey C. Bowker; and Christine L. Borgman (2011). Science Friction: Data, Metadata, and Collaboration. *Social Studies of Science,* vol. 41, no. 5, pp. 667-690. 10.1177/0306312711413314 22164720

[CR32] Faniel, Ixchel M.; and Trond E. Jacobsen (2010). Reusing Scientific Data: How Earthquake Engineering Researchers Assess the Reusability of Colleagues’ Data. *Computer Supported Cooperative Work (CSCW)*, vol. 19, no. 3–4, pp. 355–375.

[CR33] Faniel, Ixchel; R. Frank; and E. Yakel (2019). Context from the Data Reuser’ Point of View. *Journal of Documentation*, vol. 75, pp. 1275 – 1297.

[CR34] Gaia Collaboration (2021). *Gaia* Early Data Release 3: Summary of the Contents and Survey Properties. *Astronomy & Astrophysics*, vol. 649, paper A1.

[CR35] Garfinkel, Harold (1967). *Studies in Ethnomethodology*. Englewood Cliffs, New Jersey: Prentice-Hall.

[CR36] Garfinkel, Harold (2002). *Ethnomethodology's Program: Working out Durkheim’s Aphorism*. A. Rawls (ed.). Lanham, Maryland: Rowman & Littlefield.

[CR37] Garfinkel, Harold (2008). *Toward a Sociological Theory of Information*. A. Rawls (ed.). Lanham, Boulder, Colorado: Paradigm.

[CR38] Gilbert, Margaret (2000). *Sociality and Responsibility: New Essays on Plural Subject Theory*. Lanham, MD: Rowman and Littlefield.

[CR39] Goodwin, Charles (2013). The Co-operative, Transformative Organization of Human Action and Knowledge. *Journal of Pragmatics*, vol. 46, no. 1, pp. 8 – 23.

[CR40] Goodwin, Charles (2018). *Co-operative Action*. Cambridge: Cambridge University Press.

[CR41] Goody, Jack (1977). *The Domestication of the Savage Mind*. Cambridge: Cambridge University Press.

[CR42] Hacking, Ian (1992). The Self-vindication of the Laboratory Sciences. In Andrew Pickering (ed). *Science as Culture and Practice*. Chicago. Ill.: University of Chicago Press, pp. 19-64.

[CR43] Hanisch, R.J.; A. Farris; E.W. Greisen; W.D. Pence; B.M. Schlesinger; P. J. Teuben; R. W. Thompson; and A. Warnock III (2001). Definition of the Flexible Image Transport System (FITS). *Astronomy and Astrophysics*, vol. 376, pp. 359-380.

[CR44] Hanson, Norwood Russell (1958). *Patterns of Discovery*. Cambridge: Cambridge University Press.

[CR45] Hardwig, John (1991). The Role of Trust in Knowledge. *Journal of Philosophy*, vol. 88, no. 12, pp. 693-708.

[CR46] Harper, Richard H. R. (1998). *Inside the IMF: An Ethnography of Documents, Technology and Organisational Action*. San Diego: Academic Press.

[CR47] Hilgartner, Stephen (2017). *Reordering Life: Knowledge and Control in the Genomics Revolution*. Cambridge, Mass.: MIT Press.

[CR48] Hill, Richard J.; and Kathleen Stones Crittenden (eds.) (1968). *Proceedings of the Purdue Symposium on Ethnomethodology*. West Lafayette, Indiana: Institute for the Study of Social Change.

[CR49] Hoeppe, Götz (2014). Working Data Together: The Accountability and Reflexivity of Digital Astronomical Practice. *Social Studies of Science*, vol. 44, no. 2, pp. 243–270. 10.1177/0306312713509705 24941613

[CR50] Hoeppe, Götz (2018). Tensions of Accountability: Scientists, Technicians and the Ethical Life of Data Production in Astronomy. *Science as Culture*, vol. 27, no. 4, pp. 488–512.

[CR51] Hoeppe, Götz (2019a). Mediating Environments and Objects as Knowledge Infrastructure. *Computer Supported Cooperative Work (CSCW)*, vol. 28, no. 1-2, pp. 25–59.

[CR52] Hoeppe, Götz (2019b). Medium, Calculation, Play: On Digital Images in Scientific Practice. *Social Studies of Science*, vol. 49, no. 5, pp. 758–784.

[CR53] Hoeppe, Götz (2020). Members doing Ethnography? On Some Uses of Irony and Failed Translation, Witnessed in an Episode of Data Sharing in Open Science. *Ethnographic Studies*, vol. 17, no. 1, pp. 1 – 20.

[CR54] Hogg, David W.; and Dustin Lang (2008). Astronomical Imaging: The Theory of Everything. *arXiv:0810.3851v1* [astro-ph] 21 Oct 2008.

[CR55] Huchra, John P.; Lucas M. Macri; Karen L. Masters and 17 co-authors (2012). The 2MASS Redshift Survey—Description and Data Release. *Astrophysical Journal Supplement Series*, vol. 199, no. 26 (22pp).

[CR56] Huebner, Bryce; Rebecca Kukla; and Eric Winsberg (2017). Making an Author in Radically Collaborative Research. In Th. Boyer-Kassem; C, Mayo-Wilson; and M. Weisberg (eds.). *Scientific Collaboration and Collective Knowledge: New Essays*. Oxford: Oxford University Press, pp. 95–116.

[CR57] Jaschek, Carlos (1984). Data in Astronomy. *Computer Physics Communications*, vol. 33, no. 2, pp. 289–290.

[CR58] Jefferson, Gail (2004). Glossary of Transcript Symbols with an Introduction. In Gene Lerner (ed.). *Conversation Analysis: Studies from the First Generation*. Amsterdam and Philadelphia: John Benjamins, pp. 13–31.

[CR59] Jirotka, Marina; Charlotte P. Lee; and Gary M. Olson (2013). Supporting Scientific Collaboration: Methods, Tools and Concepts. *Computer Supported Cooperative Work (CSCW)*, vol. 22, nos. 4–6, pp. 667–715.

[CR60] Knorr-Cetina, Karin (1999). *Epistemic Cultures: How the Sciences Make Knowledge*. Cambridge, Mass.: Harvard University Press.

[CR61] Knuth, Donald; Tracy Larrabee; and Paul M. Roberts (1987). *Mathematical Writing*. http://jmlr.csail.mit.edu/reviewing-papers/knuth_mathematical_writing.pdf (retrieved 1 February 2020)

[CR62] Krämer, Sybille (2015). *Medium, Messenger, Transmission: An Approach to Media Philosophy*, Enns A (trans). Amsterdam: Amsterdam University Press.

[CR63] Kratz, John Ernest; and Carly Strasser (2015). Researcher Perspectives on Publication and Peer Review of Data. *PLoS ONE*, vol. 10, no.2: e0117619. 10.1371/journal.pone.0117619 PMC4338305 25706992

[CR64] Kuhn, Thomas S. (1961). The Function of Measurement in Modern Physical Science. *Isis*, vol. 52, no. 2, pp. 161-193.

[CR65] Kuhn, Thomas S. (1970). *The Structure of Scientific Revolutions*. Second edition. Chicago, Illinois: University of Chicago Press.

[CR66] Latour, Bruno; and Steve Woolgar (1986). *Laboratory Life: The Construction of Scientific Facts, 2*^*nd*^*ed*. Princeton, New Jersey: Princeton University Press.

[CR67] Lee, Charlotte P. (2007). Boundary Negotiating Artifacts: Unbinding the Routine of Boundary Objects and Embracing Chaos in Collaborative Work. *Computer Supported Cooperative Work (CSCW)*, vol. 16, no. 3, pp. 307–339.

[CR68] Leonelli, Sabina (2016). *Data-Centric Biology: A Philosophical Approach*. Chicago, Ill.: University of Chicago Press.

[CR69] Lindwall, Oskar; Gustav Lymer; and Christian Greiffenhagen (2015). The Sequential Analysis of Instruction. *Handbook of Classroom Discourse and Interaction*. Edited by Numa Markee. Hoboken, New Jersey: Wiley, pp. 142-157.

[CR70] Lynch, Michael (1991). Method: Measurement - Ordinary and Scientific Measurement as Ethnomethodological Phenomena. In: G. Button (ed.), *Ethnomethodology and the Human Sciences*. Cambridge: Cambridge University Press, pp. 77-108.

[CR71] Lynch, Michael; and Kathleen Jordan (1995). Instructed Action In, Of and As Molecular Biology. *Human Studies*, vol. 18, no. 2/3, pp. 227–244.

[CR72] Macbeth, Douglas (2003). Hugh Mehan’s *Learning Lessons* Reconsidered: On the Differences Between the Naturalistic and Critical Analysis of Classroom Discourse. *American Educational Research Journal*, vol. 40, no. 1, pp. 239-280.

[CR73] Macbeth, Douglas (2011). Understanding Understanding as an Instructional Matter. *Journal of Pragmatics*, vol. 43, no. 2, pp. 438-451.

[CR74] Martin, David; John Rooksby; and Mark Rouncefield (2007). Users as Contextual Features of Software Product Development and Testing. In *GROUP 2007: International Conference on Supporting Group Work, 4-7 November 2007, Sanibel Island, Florida.* New York: ACM Press pp. 301–310.

[CR75] Mayernik, M. (2019). Metadata Accounts: Achieving Data and Evidence in Scientific Research. *Social Studies of Science*, vol. 49, no. 5, pp. 732–757 10.1177/0306312719863494 PMC7323761 31354073

[CR76] McCray, W. Patrick (2017) The Biggest Data of All: Making and Sharing a Digital Universe. *Osiris*, vol. 32, no. 1, pp. 243 – 263.

[CR77] Mehan, Hugh (1979). *Learning Lessons: Social Organisation in the Classroom*. Cambridge, Mass.: Harvard University Press.

[CR78] Mosconi, Gaia; Qinyu Li; Dave Randall; Helena Karasti; Peter Tolmie; Jana Barutzky; Matthias Korn; and Volkmar Pipek (2019). Three Gaps in Opening Science. *Computer Supported Cooperative Work (CSCW)*, vol. 28, no. 3-4, pp. 749–789.

[CR79] Netz, Reviel (1999). *The Shaping of Deduction in Greek Mathematics: A Cognitive History*. Cambridge: Cambridge University Press.

[CR80] Nietzsche, Friedrich (2014). *Beyond Good and Evil / On the Genealogy of Morals*. Translated by Adrian Del Caro. Stanford, California: Stanford University Press.

[CR81] Novick, David G.; and Karen Ward (2006). Why Don’t People Read the Manual? *SIGDOC’06, SIGDOC '06: Proceedings of the 24th annual ACM international conference on Design of communication. Myrtle Beach, South Carolina, 18-20 October 2006*. New York: ACM Press, pp. 11-18.

[CR82] Ochsenbein, F.; P. Bauer; and J. Marcout (2000). The VizieR Database of Astronomical Catalogues. *Astronomy & Astrophysics Supplement Series*, vol. 143, no. 1, pp. 23-32.

[CR83] Paine, Drew; and Charlotte P. Lee (2021). Coordinative Entities: Forms of Organizing in Data Intensive Science. *Computer Supported Cooperative Work (CSCW)*, vol. 29, no. 3, pp. 335-380.

[CR84] Pasquetto, I.; B.M. Randles; and C.L. Borgman (2017). On the Reuse of Scientific Data. *Data Science Journal*, vol. 16, no. 8, pp. 1-9.

[CR85] Peirce, Charles Sanders. 1992 [1877]. The Fixation of Belief. In Nathan Houser; and Christian Kloesel, eds. *The Essential Peirce: Selected Philosophical Writings*, Volume I (1867-1893), pp. 109-23. Bloomington: Indiana University Press.

[CR86] Pepe, A.; A. Goodman; A. Muench; M. Crosas; C. Erdmann (2014). How Do Astronomers Share Data? Reliability and Persistence of Datasets Linked in AAS Publications and a Qualitative Study of Data Practices among US Astronomers. *PLoS ONE*, vol. 9, no. 8: e104798. 10.1371/journal.pone.0104798 10.1371/journal.pone.0104798 PMC4148308 25165807

[CR87] Pinch, Trevor J. (1985). Towards an Analysis of Scientific Observation: The Externality and Evidential Significance of Observational Reports in Physics. *Social Studies of Science*, vol. 15, no. 1, pp. 3-36.

[CR88] Plant, Anne L.; and Robert J. Hanisch (2020). Reproducibility in Science: A Metrology Perspective. *Harvard Data Science Review*, Issue 2: 4, pp. 1 – 28. 10.1162/99608f92.eb6ddee4

[CR89] Randall, Dave; Richard Harper; Mark Rouncefield (2007). *Fieldwork for Design: Theory and Practice*. London: Springer Verlag.

[CR90] Rheinberger, Hans-Jörg (2011). Infra-Experimentality: From Traces to Data, from Data to Patterning Facts. *History of Science*, vol. 49, no. 3, pp. 337-348.

[CR91] Rogers, Silvia M. (2014). *Mastering Scientific and Medical Writing*. Berlin: Springer-Verlag.

[CR92] Rolland, B.; and Lee, C. P. (2013). Beyond Trust and Reliability: Reusing Data in Collaborative Cancer Epidemiology Research. In *CSCW '13: Proceedings of the 2013 Conference on Computer Supported Cooperative Work*, San Antonio, Texas, USA. New York, NY: ACM, pp. 435–444.

[CR93] Rooksby, John; Mark Rouncefield; and Ian Sommerville (2009). Testing in the Wild: The Social and Organisational Dimensions of Real World Practice. *Computer Supported Cooperative Work (CSCW)*, vol. 18, no. 5-6, pp. 559–580.

[CR94] Rouse, Joseph (2003). Kuhn’s Philosophy of Scientific Practice. In: Nickles, Thomas (ed.) *Thomas Kuhn*. Cambridge: Cambridge University Press, pp. 101–121.

[CR95] Ryle, Gilbert (1949). *The Concept of Mind*. London: Hutchinson.

[CR97] Sacks, Harvey; Emanuel Schegloff; and Gail Jefferson (1974). A simplest systematics for the organization of turn-taking for conversation. *Language*, vol. 50, pp. 696-735.

[CR98] Schegloff, Emanuel A. (1991). Conversation Analysis and Socially Shared Cognition. In Lauren B. Resnick; John M. Levine; and Stephanie D. Teasley (eds.). *Perspectives on Socially Shared Cognition*. Washington, DC: American Psychological Association, pp. 150-171.

[CR99] Schegloff, Emanuel A. (1992). Repair After Next Turn: The Last Structurally Provided Place for the Defense of Intersubjectivity in Conversation. *American Journal of Sociology* 95(5): 1295-1345.

[CR100] Schmidt, Kjeld (2011). *Cooperative Work and Coordinative Practices*. London: Springer Verlag.

[CR101] Schmidt, Kjeld; and Liam J. Bannon (1992). Taking CSCW Seriously: Supporting Articulation Work. *Computer Supported Cooperative Work (CSCW)*, vol. 1, nos. 1–2, pp. 7–40.

[CR102] Schmidt, Kjeld; and Carla Simone (1996). Coordination Mechanisms: Towards a Conceptual Foundation of CSCW Systems Design. *Computer Supported Cooperative Work (CSCW)*, vol. 5, no. 2–3, pp. 155–200.

[CR103] Schmidt, Kjeld; and Ina Wagner (2004). Ordering Systems: Coordinative Practices and Artifacts in Architectural Design and Planning. *Computer Supported Cooperative Work (CSCW)*, vol. 13, no. 3–4, pp. 349–408.

[CR104] Shah, Hetan (2018). Algorithmic Accountability. *Philosophical Transactions of the Royal Society A* 376: 20170362. 10.1098/rsta.2017.0362 10.1098/rsta.2017.0362 30082307

[CR105] Shannon, Claude; and Warren Weaver (1964). *The Mathematical Theory of Communication*. Urbana Ill.: University of Illinois Press.

[CR106] Sharrock, Wes; and R.J. Anderson (1994). The User as a Scenic Feature of the Design Space. *Design Studies*, vol. 15, no. 1, pp. 5 – 18.

[CR107] Smith, Dorothy E. (2001). Texts and the Ontology of Organizations and Institutions. *Studies in Cultures, Organizations and Societies* 7 (2): 159 – 198.

[CR108] Spencer, Matt (2015). Brittleness and Bureaucracy: Software as a Material for Science. *Perspectives on Science*, vol. 23, no. 4, pp. 466-484.

[CR109] Steinhardt, Stephanie B.; and Steven J. Jackson (2014). Reconciling Rhythms: Plans and Temporal Alignment in Collaborative Scientific Work. In *CSCW 2014: Proceedings of the 17th ACM Conference on Computer Supported Cooperative Work & Social Computing.* *Baltimore, 15-19 February 2014*. New York: ACM Press, pp.134-145.

[CR110] Stoughton, Chris; and 191 co-authors (2002). Sloan Digital Sky Survey: Early Data Release. *Astronomical Journal*, vol. 123, pp. 485-548.

[CR111] Strauss, Anselm (1988). The Articulation of Project Work: An Organizational Process. *Sociological Quarterly*, vol. 29, no. 2, pp. 163-178.

[CR112] Suchman, Lucy (2007). *Human-Computer Reconfigurations*. Cambridge: Cambridge University Press.

[CR113] Tenopir, Carol; Suzie Allard; Kimberly Douglass; Arsev U. Aydinoglu; Lei, Wu; Eleanor Read; Maribeth Manoff; and Mike Frame (2011). Data Sharing by Scientists: Practices and Perceptions. *PLoS ONE*, vol. 6, no. 6: e21101. 10.1371/journal.pone.0021101 PMC3126798 21738610

[CR114] Tenopir, C.; E. Dalton; S. Allard; M. Frame; I. Pjesivac; B. Birch; D. Pollock; and K. Dorsett (2015). Changes in Data Sharing and Data Reuse Practices and Perceptions among Scientists Worldwide. *PLoS One*, vol. 10, no. 8: e0134826. 10.1371/journal.pone.0134826 PMC4550246 26308551

[CR115] van de Sandt, S.; S. Dallmeier-Tiessen; A. Lavasa; and V. Petras (2019). The Definition of Reuse. *Data Science Journal*, 18: 22, pp. 1–19.

[CR116] Wagenknecht, Susann (2016). *A Social Epistemology of Research Groups: Collaboration in Scientific Practice*. London: Palgrave Macmillan.

[CR117] Whittaker, J. (2002). *How to Break Software: A Practical Guide to Testing*. Boston: Addison-Wesley.

[CR118] Wilkinson, M. D. et al. (2016). The FAIR Guiding Principles for Scientific Data Management and Stewardship. *Scientific Data* 3:160018 doi: 10.1038/sdata.2016.18. 10.1038/sdata.2016.18 PMC4792175 26978244

[CR119] Wimsatt, William (2012). Robustness, Reliability and Overdetermination. In *Characterizing the Robustness of Science*. Soler, L.; Trizio, E.; Nickles, T.; and Wimsatt, W. (eds). Dordrecht: Springer, pp. 61-87.

[CR120] Wittgenstein, Ludwig (2009). *Philosophical Investigations*. 4^th^ edition. Oxford: Blackwell.

[CR121] Woolgar, Steve (1991). Configuring the User: The Case of Usability Trials. In Law, J. (ed.). *A Sociology of Monsters: Essays on Power Technology and Domination*. London: Routledge, pp. 58-100.

[CR122] Wray, K. Brad (2007). Who Has Scientific Knowledge? *Social Epistemology*, vol. 21, no. 3, pp. 337-347.

[CR123] Wray, K. Brad (2017). The Impact of Collaboration on the Epistemic Cultures of Science. In Th. Boyer-Kassem; C. Mayo-Wilson; and M. Weisberg (eds.). *Scientific Collaboration and Collective Knowledge: New Essays*. Oxford: Oxford University Press, pp. 117-134.

[CR124] Wulf, William (1993). The Collaboratory Opportunity. *Science*, vol. 261, pp. 854–855. 10.1126/science.8346438 8346438

[CR125] Yakel, Elizabeth; Ixchel M. Faniel; and Zachary J. Maiorana (2019). Virtuous and Vicious Circles in the Data Life-cycle. *Information Research*, vol. 24, no. 2, paper 821.

[CR126] Yoon, Ayoung (2016). Red Flags in Data: Learning from Failed Data Reuse Experiences. *ASIS&T Annual Meeting Proceedings*, vol. 53, no. 1, pp. 1-6.

[CR127] York, Donald G. et al. (2000). The Sloan Digital Sky Survey: Technical Summary. *Astronomical Journal*, vol. 120, pp. 1679-1588.

[CR128] Zimmerman, Anne (2007). Not By Metadata Alone: The Use of Diverse Forms of Knowledge to Locate Data for Reuse. *International Journal on Digital Libraries*, vol. 7, no. 1–2, pp. 5–16.

